# Confidence bands for multiplicative hazards models: Flexible resampling approaches

**DOI:** 10.1111/biom.13059

**Published:** 2019-04-17

**Authors:** Dennis Dobler, Markus Pauly, ThomasH. Scheike

**Affiliations:** ^1^ Department of Mathematics Vrije Universiteit Amsterdam Amsterdam Netherlands; ^2^ Faculty of Statistics Technical University of Dortmund Dortmund Germany; ^3^ Section of Biostatistics University of Copenhagen Copenhagen Denmark

**Keywords:** competing risks, counting processes, Cox regression, hazards, Martingale theory, multistate models, survival analysis

## Abstract

We propose new resampling‐based approaches to construct asymptotically valid time‐simultaneous confidence bands for cumulative hazard functions in multistate Cox models. In particular, we exemplify the methodology in detail for the simple Cox model with time‐dependent covariates, where the data may be subject to independent right‐censoring or left‐truncation. We use simulations to investigate their finite sample behavior. Finally, the methods are utilized to analyze two empirical examples with survival and competing risks data.

## 1 INTRODUCTION

Multiplier resampling (also known as the wild bootstrap) has evolved as a state‐of‐the‐art choice for inference on cumulative incidences or hazards in nonparametric and semiparametric survival and multistate models in event history analysis; see Lin et al. (1993) and Lin et al. (1994) for Cox models and Lin (1997) for competing risks set‐ups. The basic idea is to mimic martingale representations of the estimators by replacing nonobservable martingale residuals with randomly weighted counting processes. This approach has been extended in various directions, allowing for a greater flexibility (Beyersmann et al., 2013; Dobler & Pauly, 2014; Dobler et al., 2017) and multiple, possibly recurrent, states (Bluhmki et al., 2018, 2019). We will here focus on semiparametric regression models. The most used regression model in survival analysis is Cox's proportional hazard model (Cox, 1972). It is highly useful to predict survival probabilities based on covariates. Such survival functions are typically provided with point‐wise confidence intervals, and this is implemented in all major software packages. However, when interest is in the survival function as a whole, it is preferable to report it together with uniform confidence intervals. These so‐called confidence bands describe the uncertainty of the whole survival function estimation. But this is infrequently done in practice because there are few programs that construct such uniform bands. In addition, apart from only few exceptions such as Lin (1997), systematic evaluations of finite sample results with demonstrations of the bands’ performance are rarely available in the literature. In the present article we provide such results in Cox models and we investigate various new resampling bands that exhibit a reliable performance even for small sample sizes.

Our main achievements are the introduction of resampling strategies that jointly mimic the unknown distribution of baseline and parameter estimators in Cox models and corresponding multistate versions. We provide theoretical justification for the resampling approaches based on martingale arguments, thereby allowing for different mechanisms of incomplete observations and simplifying many arguments.

There exist many resampling approaches for the Cox model with independent right‐censoring. One approach is based on the martingale representations of the Breslow estimator for the cumulative hazard and the parameter estimator, where all unobservable martingale residuals $dM_{i}\left(u\right)$ are replaced with reweighted counting processes $G_{i}dN_{i}\left(u\right)$ (e.g., Lin et al., 1994). Another possibilty that works more generally, for example for rate estimation, is to replace the martingale instead with $G_{i}d{\hat{M}}_{i}\left(u\right)$ (e.g., Spiekerman & Lin, 1998). This exploits the model's semiparametric structure more actively.

We will also use a more natural approach, which starts one step earlier by rewriting the score equations for the baseline function and the Euclidean parameter: we apply the multiplier techniques from above to martingale representations of the score equations. This leads to new equations, which are solved by quantities depending on the multipliers. Hence, paralleling the same steps as for the original estimators, their resampling counterparts are obtained in an elementary way. Throughout the article, we follow the flexible approach of Beyersmann et al. (2013) and allow for general multiplier distributions.

For ease of presentation, we exemplify the new methodology mainly for the Cox model in a survival setting but we also discuss extensions to more general multistate and other regression models. We are able to base our theoretical derivations for the resampling approaches on martingale arguments, which are novel in the semiparametric context. Thus, intricate derivations for verifying conditional tightness, that is, tightness of the resampling process conditioned on the observed data, are no longer required and are handled conveniently by the martingale central limit theorems. The martingale arguments for the multiplier bootstrap are motivated by those for resampling nonparametric Aalen‐Johansen estimators (Bluhmki et al., 2018). In particular, we prove similar martingale properties of the resampling counterparts as for the original estimators. Moreover, mirroring the martingale structure in the bootstrap world allows for a simple interpretation and easy incorporation of missingness mechanisms, for example, independent right‐censoring or left‐truncation. To conclude, our findings allow for a wide range of applications some of which will be discussed in more detail in future papers.

The paper is organized as follows: Section 2 sketches how estimators in Cox models are found and it lists the technical conditions required for all of their large sample properties. Section 3 contains a derivation of all considered multiplier bootstrap procedures and also theoretical statements about their validity. In addition, we discuss several important extensions to more general multistate or other regression models. In Section 4, we compare the resampling procedures in an extensive simulation study. Section 5 has a brief demonstration of the methodology in a survival setting, where interest is on constructing confidence bands for the survival function for patients with acute myocardial infarction, and another demonstration of confidence bands for cumulative incidence functions for patients after a bone marrow transplantation. Finally, we discuss the findings in Section 6.

The online supporting information contains all proofs whose novelty lies in the considerable simplification of the technical arguments by transferring martingale methods to the resampling procedures in the present semiparametric framework. It also contains a second small simulation study, algorithmic descriptions of the multiplier bootstraps, and links to GitHub pages with example R code for user‐friendly applicability of our procedures.

## 2 JOINT LARGE SAMPLE PROPERTIES IN THE COX MODEL

We consider multiplicative *Cox* (or *proportional hazards*) models given by the intensity

(1) $$\lambda_{i}\left(t,\beta_{0}\right)=Y_{i}\left(t\right)\lambda_{0}\left(t\right)\text{exp}\left\{X\prime_{i}\left(t\right)\beta_{0}\right\}.$$

of the counting process $N_{i}\left(t\right)$ of subject i=1,…,n, possibly subject to independent left‐truncation and/or right‐censoring, given its possibly time‐dependent p‐dimensional vector of predictable covariates $X_{i}\left(t\right)=\left\{X_{i1}\left(t\right),\ldots ,X_{ip}\left(t\right)\right\}\prime$. That is, $E\left\{dN_{i}\left(t\right)|X_{i}\left(t\right)\right\}=\lambda_{i}\left(t,\beta_{0}\right)dt$. Here, $N_{i}\left(t\right)\in \left\{0,1\right\}$ counts the occurence of a particular event for subject i not later than treatment time $t,Y_{i}\left(t\right)$ is the corresponding at‐risk indicator at that time, $\lambda_{0}$ is the baseline hazard function, and $\beta_{0}=\left(\beta_{1},\ldots ,\beta_{p}\right)\prime \in R^{p}$ is an unknown regression parameter (Cox, 1972). Let τ>0 be a terminal evaluation time on the treatment time‐scale. Throughout we assume all $X_{i}\left(t\right)$ to be contained in a bounded set and that the cumulative baseline hazard $\Lambda_{0}\left(t\right)=\int_{0}^{t}\lambda_{0}\left(s\right)ds$ is finite at t=τ. Standard arguments yield the *Breslow estimator* ${\hat{\Lambda }}_{0}$ of $\Lambda_{0}$ and the maximum partial likelihood parameter estimator $\hat{\beta }$ of $\beta_{0}$. To illustrate this, we simplify derivations of Scheike and Zhang (2002) for Cox‐Aalen models to the Cox model Equation (1): the score equation for $\Lambda_{0}$ is

(2) $$\sum_{i=1}^{n}\left[dN_{i}\left(t\right)-Y_{i}\left(t\right)\text{exp}\left\{X\prime_{i}\left(t\right)\beta \right\}d\Lambda_{0}\left(t\right)\right]=0,$$

and solved by ${\hat{\Lambda }}_{0}\left(t,\beta \right)=\sum_{i=1}^{n}\int_{0}^{t}J\left(u\right)S_{0}^{-1}\left(u,\beta \right)dN_{i}\left(u\right)$. We will make use of the definitions

$$S_{k}\left(t,\beta \right)=\sum_{i=1}^{n}X_{i}^{⊗k}\left(t\right)Y_{i}\left(t\right)\text{exp}\left\{X\prime_{i}\left(t\right)\beta \right\},\;k=0,1,2,$$

where $y^{⊗2}=yy\prime \in R^{p\times p},y^{⊗1}=y\in R^{p}$ and $y^{⊗0}=1\in R$ for any vector $y\in R^{p}$, and J(u) is the indicator that any individual is under risk shortly before u. For simplicity, the notion of J(u) is usually suppressed. Replacing ${\hat{\Lambda }}_{0}\left(t,\beta \right)$ for $\Lambda_{0}$ in the score equation for β, that is,

(3) $$\sum_{i=1}^{n}\int_{0}^{\tau }X_{i}\left(u\right)Y_{i}\left(u\right)\left[dN_{i}\left(u\right)-\text{exp}\left\{X\prime_{i}\left(u\right)\beta \right\}d\Lambda_{0}\left(u\right)\right]=0,$$

and defining $E\left(t,\beta \right)=S_{1}\left(t,\beta \right)S_{0}^{-1}\left(t,\beta \right)$, we obtain a solvable score equation for β:

$$U_{\tau }\left(\beta \right)=\sum_{i=1}^{n}\int_{0}^{\tau }\left\{X_{i}\left(u\right)-E\left(u,\beta \right)\right\}dN_{i}\left(u\right)=0.$$

Through its solution $\hat{\beta }$ we obtain the *Breslow estimator* ${\hat{\Lambda }}_{0}\left(t,\hat{\beta }\right)$ of $\Lambda_{0}\left(t\right)$. The central limit theorem for $\hat{\beta }$ stated below requires the negative Jacobian of $U_{\tau }$ with respect to β, that is, $I_{\tau }\left(\beta \right)=-DU_{\tau }\left(\beta \right)=\int_{0}^{\tau }V\left(u,\beta \right)dN\left(u\right)$, where $V\left(t,\beta \right)=S_{2}\left(t,\beta \right)S_{0}^{-1}\left(t,\beta \right)-E^{⊗2}\left(t,\beta \right)$ and $N=\sum_{i=1}^{n}N_{i}$. Due to the assumed boundedness of the covariates, it follows from Theorems VII.2.2 (p. 498) and VII.2.3 (p. 503f.) in Andersen et al. (1993) that $\sqrt{n}\left(\hat{\beta }-\beta_{0}\right)$ and $\sqrt{n}\left\{{\hat{\Lambda }}_{0}\left(\cdot ,\hat{\beta }\right)-\Lambda_{0}\left(\cdot \right)\right\}$ are asymptotically Gaussian provided the following regularity conditions hold; see Condition VII.2.1 in Andersen et al. (1993, p. 497). Let $\overset{p}{\to }$ denote convergence in probability as n→∞ and ∇ the gradient operator with respect to β.

Condition 1 There exists a neighborhood B of $\beta_{0}$ and functions $s_{0}:\left[0,\tau \right]\times B\to R,s_{1}:\left[0,\tau \right]\times B\to R^{p}$, and $s_{2}:\left[0,\tau \right]\times B\to R^{p\times p}$ such that for each k=0,1,2:

(a) ${\text{sup}}_{\beta \in B,t\in \left[0,\tau \right]}∥n^{-1}S_{k}\left(t,\beta \right)-s_{k}\left(t,\beta \right)∥\overset{p}{\to }0$, where ∥⋅∥ denotes the Euclidean norm;
(b) $s_{k}$ is a continuous function of β∈B uniformly in t∈[0,τ] and bounded on [0,τ]×B;
(c) $s_{0}\left(\cdot ,\beta_{0}\right)$ is bounded away from zero on [0,τ];
(d) $s_{2}\left(t,\beta \right)=Ds_{1}\left(t,\beta \right)$ and $s_{1}\left(t,\beta \right)=\left\{\nabla s_{0}\left(t,\beta \right)\right\}\prime$ for β∈B,t∈[0,τ];
(e) ${\mathrm{Sigma}}_{\tau }=\int_{0}^{\tau }v\left(t,\beta_{0}\right)s_{0}\left(t,\beta_{0}\right)d\Lambda_{0}\left(t\right)$ is positive definite, where $v\left(t,\beta \right)=s_{2}\left(t,\beta \right)s_{0}^{-1}\left(t,\beta \right)-{\left\{s_{1}\left(t,\beta \right)s_{0}^{-1}\left(t,\beta \right)\right\}}^{⊗2}$.

Throughout, we assume Condition 1 to be satisfied. Note that (a) and (b) immediately imply

(4) $$\underset{t\in \left[0,\tau \right]}{\sup}∥S_{k}\left(t,\tilde{\beta }\right)-s_{k}\left(t,\beta_{0}\right)∥\overset{p}{\to }0$$

for each k=0,1,2 as long as $\tilde{\beta }\overset{p}{\to }\beta_{0}$. The proofs of the aforementioned theorems in Andersen et al. (1993) make use of the following asymptotic representations of the estimators:

(5) $$\sqrt{n}\left(\hat{\beta }-\beta_{0}\right)={\left\{\frac{1}{n}I_{\tau }\left(\beta_{0}\right)\right\}}^{-1}\frac{1}{\sqrt{n}}U_{\tau }\left(\beta_{0}\right)+o_{p}\left(1\right)$$

(6) $$\sqrt{n}\left\{{\hat{\Lambda }}_{0}\left(\cdot ,\hat{\beta }\right)-\Lambda_{0}\left(\cdot \right)\right\}=-\sqrt{n}\left(\hat{\beta }-\beta_{0}\right)\prime \int_{0}^{\cdot }e\left(u,\beta_{0}\right)d\Lambda_{0}\left(u\right)+\sqrt{n}\int_{0}^{\cdot }\frac{dM\left(u\right)}{S_{0}\left(u,\beta_{0}\right)}+o_{p}\left(1\right).$$

These representations motivate the first bootstrap approach in Section 3. Here, e is the probability limit of E, and $M\left(t\right)=\sum_{i=1}^{n}M_{i}\left(t\right)=\sum_{i=1}^{n}\left\{N_{i}\left(t\right)-\int_{0}^{t}\lambda_{i}\left(u,\beta_{0}\right)du\right\}$ is a square‐integrable martingale in t∈[0,τ]; cf. Andersen et al. (1993, Section VII.2.2; p. 496ff) .

## 3 MULTIPLIER RESAMPLING APPROACHES AND MAIN THEOREMS

While inference about $\beta_{0}$ can be based on the asymptotic normality of its estimator (e.g., Martinussen & Scheike, 2006; p. 184ff.), the complicated limit process of the normalized Breslow estimator hinders straightforward time‐simultaneous inference about $\Lambda_{0}$ or functionals thereof, for example, the survival function. As a remedy we will investigate two resampling strategies, both of which make use of independent i.i.d. zero mean and unit variance random variables $G_{1},\ldots ,G_{n}$, which are henceforth called *multipliers*. Since ${\hat{\Lambda }}_{0}$ and $\hat{\beta }$ are dependent, we have to ensure that the bootstrap procedures respect their dependence structure.

### 3.1 The ‘Classical’ multiplier bootstrap

The *first method* is inspired by the resampling procedures of Lin et al. (1994) and Spiekerman and Lin (1998). They use standard normal multipliers, which is motivated from the asymptotic martingale representation of the normalized estimators. The idea is to replace the martingale increments $dM_{i}\left(t\right)=dN_{i}\left(t\right)-Y_{i}\left(t\right)\text{exp}\left\{X\prime_{i}\left(t\right)\beta_{0}\right\}d\Lambda_{0}\left(t\right)$ with $G_{i}dN_{i}\left(t\right)$ (Lin et al., 1994) or with $G_{i}{\hat{M}}_{i}\left(t\right)=G_{i}\left[dN_{i}\left(u\right)-Y_{i}\left(u\right)\text{exp}\left\{X\prime_{i}\left(u\right)\hat{\beta }\right\}d{\hat{\Lambda }}_{0}\left(u,\hat{\beta }\right)\right]$, using estimated martingale increments (Spiekerman & Lin, 1998). We extend these approaches in several ways: our approaches are not restricted to normal multipliers $G_{i}$; cf. Beyersmann et al. (2013) where centered Poisson multipliers exhibited a better finite sample behavior in a nonparametric setting. Also, to be in line with the usual guidelines for bootstrap‐based inference (Hall & Wilson, 1991), we provide and analyze suitable bootstrap versions of the covariance estimators. Finally, in Section 3.4 an extension to more general models is outlined.

We exemplify the general resampling idea for $G_{i}dN_{i}\left(u\right)$ by introducing multiplier bootstrap versions of the score equation defining vector $U_{\tau }$ and its negative Jacobian $I_{\tau }$.

(7) $$U_{\tau }^{*}\left(\hat{\beta }\right)=\sum_{i=1}^{n}G_{i}\int_{0}^{\tau }\left\{X_{i}\left(t\right)-E\left(t,\hat{\beta }\right)\right\}dN_{i}\left(t\right)\;\text{and}$$

(8) $$\frac{1}{n}I_{\tau }^{*}\left(\hat{\beta }\right)=\frac{1}{n}\sum_{i=1}^{n}G_{i}^{2}\int_{0}^{\tau }\left\{X_{i}\left(t\right)-E\left(t,\hat{\beta }\right)\right\}\left\{X_{i}\left(t\right)-E\left(t,\hat{\beta }\right)\right\}\prime dN_{i}\left(t\right).$$

Here and below, all objects with asterisks refer to bootstrap quantities. Following the asymptotic representation Equations (5) and (6), we find their following multiplier bootstrap counterparts:

(9) $$\sqrt{n}\left({\hat{\beta }}^{*}-\hat{\beta }\right)\;=\;{\left\{\frac{1}{n}I_{\tau }^{*}\left(\hat{\beta }\right)\right\}}^{-1}\frac{1}{\sqrt{n}}U_{\tau }^{*}\left(\hat{\beta }\right),$$

(10) $$\sqrt{n}\left\{{\hat{\Lambda }}_{0}^{*}\left(\cdot ,{\hat{\beta }}^{*}\right)-{\hat{\Lambda }}_{0}\left(\cdot ,\hat{\beta }\right)\right\}=-\sqrt{n}\left({\hat{\beta }}^{*}-\hat{\beta }\right)\prime \int_{0}^{\cdot }E\left(u,\hat{\beta }\right){\hat{\Lambda }}_{0}\left(du,\hat{\beta }\right)+\sqrt{n}\sum_{i=1}^{n}G_{i}\int_{0}^{\cdot }\frac{dN_{i}\left(u\right)}{S_{0}\left(u,\hat{\beta }\right)}.$$

Note that Equations (9) and (10) are essentially definitions of ${\hat{\beta }}^{*}$ and ${\hat{\Lambda }}_{0}^{*}\left(\cdot ,{\hat{\beta }}^{*}\right)$, respectively, and that the $o_{p}\left(1\right)$ terms in Equations (5) and (6) have been dropped. Alternatively to this multiplier bootstrap choice, the Spiekerman and Lin (1998)‐type martingale increment estimates $G_{i}d{\hat{M}}_{i}\left(u\right)$ may replace $G_{i}dN_{i}\left(u\right)$ in Equations (7) and (10). A bootstrap‐type covariance estimate similar to Equation (8) has been suggested by Dobler and Pauly (2014) in a nonparametric competing risks context. Here, it is additionally motivated from martingale arguments: defining $I_{t}^{*}$ and $U_{t}^{*}$ as in Equations (7) and (8) but t replacing τ, we found that ${\left\{I_{t}^{*}\left(\hat{\beta }\right)\right\}}_{t\in \left[0,\tau \right]}$ is the optional variation process of the square‐integrable *martingale* ${\left\{n^{-1∕2}U_{t}^{*}\left(\hat{\beta }\right)\right\}}_{t\in \left[0,\tau \right]}$; see the supporting information for details.

The next subsection offers an alternative resampling strategy, which does not ignore the $o_{p}\left(1\right)$ terms in Equations (5) and (6). Instead, they are naturally incorporated into the resampling step.

### 3.2 Bootstrapping the score equations

The idea of the *second* approach is to replace martingale representations of *score equations* with their multiplier counterparts. To this end, we parallel the classic approach of jointly solving two score equations and first expand the score equation in Equation (2) to $\sum_{i=1}^{n}dM_{i}\left(t\right)+\sum_{i=1}^{n}Y_{i}\left(t\right)\left[\text{exp}\left\{X\prime_{i}\left(t\right)\beta_{0}\right\}-\text{exp}\left\{X\prime_{i}\left(t\right)\beta \right\}\right]d\Lambda_{0}\left(t\right)=0$. A multiplier bootstrap counterpart thereof is now given by replacing $dM_{i}\left(t\right)$ with $G_{i}dN_{i}\left(t\right),\beta_{0}$ with $\hat{\beta }$, and $\Lambda_{0}\left(t\right)$ with ${\hat{\Lambda }}_{0}\left(t,\hat{\beta }\right)$:

(11) $$\begin{array}{c} \sum_{i=1}^{n}G_{i}dN_{i}\left(t\right)+\sum_{i=1}^{n}Y_{i}\left(t\right)\left[\text{exp}\left\{X\prime_{i}\left(t\right)\hat{\beta }\right\}-\text{exp}\left\{X\prime_{i}\left(t\right)\beta \right\}\right]d{\hat{\Lambda }}_{0}\left(t,\hat{\beta }\right)\\ =\sum_{i=1}^{n}\left(G_{i}+1\right)dN_{i}\left(t\right)-S_{0}\left(t,\beta \right)d{\hat{\Lambda }}_{0}\left(t,\hat{\beta }\right)\overset{!}{=}0. \end{array}$$

Keeping β fixed, its “solution” for ${\hat{\Lambda }}_{0}\left(t,\hat{\beta }\right)$ is

(12) $${\hat{\Lambda }}_{0}^{*}\left(t,\beta \right)=\sum_{i=1}^{n}\left(G_{i}+1\right)\int_{0}^{t}\frac{dN_{i}\left(t\right)}{S_{0}\left(t,\beta \right)}.$$

Next, consider a martingale representation of the score Equation (3) for β:

$$\sum_{i=1}^{n}\int_{0}^{\tau }X_{i}\left(t\right)Y_{i}\left(t\right)dM_{i}\left(t\right)+\sum_{i=1}^{n}\int_{0}^{\tau }X_{i}\left(t\right)Y_{i}\left(t\right)\left[\text{exp}\left\{X\prime_{i}\left(t\right)\beta_{0}\right\}-\text{exp}\left\{X\prime_{i}\left(t\right)\beta \right\}\right]d\Lambda_{0}\left(t\right)=0.$$

This motivates a multiplier resampling version given by

$$\begin{array}{c} \sum_{i=1}^{n}G_{i}\int_{0}^{\tau }X_{i}\left(t\right)Y_{i}\left(t\right)dN_{i}\left(t\right)+\sum_{i=1}^{n}\int_{0}^{\tau }X_{i}\left(t\right)Y_{i}\left(t\right)\left[\text{exp}\left\{X\prime_{i}\left(t\right)\hat{\beta }\right\}-\text{exp}\left\{X\prime_{i}\left(t\right)\beta \right\}\right]d{\hat{\Lambda }}_{0}\left(t,\hat{\beta }\right)\\ =\sum_{i=1}^{n}\int_{0}^{\tau }\left\{X_{i}\left(t\right)Y_{i}\left(t\right)G_{i}+E\left(t,\hat{\beta }\right)\right\}dN_{i}\left(t\right)-\int_{0}^{\tau }S_{1}\left(t,\beta \right)d{\hat{\Lambda }}_{0}\left(t,\hat{\beta }\right)\overset{!}{=}0. \end{array}$$

Inserting ${\hat{\Lambda }}_{0}^{*}\left(t,\beta \right)$ for ${\hat{\Lambda }}_{0}\left(t,\hat{\beta }\right)$ eventually yields the final *multiplier bootstrap score equation*

(13) $$\begin{array}{ccc} U_{\tau }^{*}\left(\beta \right) & = & \sum_{i=1}^{n}\int_{0}^{\tau }\left\{X_{i}\left(t\right)Y_{i}\left(t\right)G_{i}+E\left(t,\hat{\beta }\right)\right\}dN_{i}\left(t\right)-\int_{0}^{\tau }E\left(t,\beta \right)\sum_{i=1}^{n}\left(G_{i}+1\right)dN_{i}\left(t\right)\\ & = & \sum_{i=1}^{n}\left(G_{i}+1\right)\int_{0}^{\tau }\left\{X_{i}\left(t\right)-E\left(t,\beta \right)\right\}dN_{i}\left(t\right)\overset{!}{=}0. \end{array}$$

The last equality is due to $\sum_{i=1}^{n}\int_{0}^{\tau }\left\{X_{i}\left(t\right)-E\left(t,\hat{\beta }\right)\right\}dN_{i}\left(t\right)=U_{\tau }\left(\hat{\beta }\right)=0$. Hence, $U_{\tau }^{*}\left(\hat{\beta }\right)$ coincides with formula Equation (7). Define ${\hat{\beta }}^{*}$ as the solution to Equation (13). Similarly as in the proof of Theorem VII.2.1 in Andersen et al. (1993, p. 497f.), it can be shown that the probability of the existence of tends to one and that ${\hat{\beta }}^{*}-\hat{\beta }$ converges to zero in probability as n→∞; see also the proof of Theorem 1 below for similar arguments.

Finally, we combine Equation (12) with ${\hat{\beta }}^{*}$ to find a resampling version of the Breslow estimator, denoted ${\hat{\Lambda }}_{0}^{*}\left(\cdot ,{\hat{\beta }}^{*}\right)$. After centering it at ${\hat{\Lambda }}_{0}\left(t,\hat{\beta }\right)$ and multiplying it with $\sqrt{n}$, it equals

(14) $$\begin{array}{c} \sqrt{n}\sum_{i=1}^{n}\left(G_{i}+1\right)\int_{0}^{t}\left\{S_{0}^{-1}\left(u,{\hat{\beta }}^{*}\right)-S_{0}^{-1}\left(u,\hat{\beta }\right)\right\}dN_{i}\left(u\right)+\sqrt{n}\sum_{i=1}^{n}G_{i}\int_{0}^{t}\frac{dN_{i}\left(u\right)}{S_{0}\left(u,\hat{\beta }\right)}\\ =\sqrt{n}\sum_{i=1}^{n}\int_{0}^{t}\left\{S_{0}^{-1}\left(u,{\hat{\beta }}^{*}\right)-S_{0}^{-1}\left(u,\hat{\beta }\right)\right\}dN_{i}\left(u\right)+\sqrt{n}\sum_{i=1}^{n}G_{i}\int_{0}^{t}\frac{dN_{i}\left(u\right)}{S_{0}\left(u,{\hat{\beta }}^{*}\right)}. \end{array}$$

A Taylor expansion around $\hat{\beta }$ of the first term on the right‐hand side reveals the striking similarity to decomposition Equation (10). However, the current multiplier bootstrap approach does not ignore the $o_{p}\left(1\right)$ term resulting from the Taylor expansion. Another nice property of this “estimating equation” approach is the similar treatment for bootstrap and original estimator, which is in line with general recommendations for constructing resampling algorithms (Efron & Tibshirani, 1994). We are going to use the mean value theorem (cf. Feng et al., 2013) to analyze the asymptotic behavior of ${\hat{\beta }}^{*}$. To this end, we introduce a modified Jacobian operator. For a continuously differentiable function $H=\left(H_{1},\ldots ,H_{p}\right)\prime :R^{p}\to R^{p}$ and a matrix $B=\left(\beta^{\left(1\right)}|\ldots |\beta^{\left(p\right)}\right)\in R^{p\times p}$ we define $\bar{D}H\left(B\right)=\left(\nabla H_{1}\left(\beta^{\left(1\right)}\right)\prime ,\ldots ,\nabla H_{p}\left(\beta^{\left(p\right)}\right)\prime \right)\prime .$ The mean value theorem yields that $-U_{\tau }^{*}\left(\hat{\beta }\right)=U_{\tau }^{*}\left({\hat{\beta }}^{*}\right)-U_{\tau }^{*}\left(\hat{\beta }\right)=\bar{D}U_{\tau }^{*}\left(\tilde{B}\right)\left({\hat{\beta }}^{*}-\hat{\beta }\right)$, where every column of $\tilde{B}$ is on the line segment between ${\hat{\beta }}^{*}$ and $\hat{\beta }$.

### 3.3 Consistency and confidence bands for the cumulative hazard

The validity proof of both resampling strategies from Sections 3.1 and 3.2 is based on

Lemma 1 Under Conditions 1(a)‐(e) it holds that, as n→∞

(I) $∥{\hat{\beta }}^{*}-\hat{\beta }∥\overset{p}{\to }0$,
(II) $-n^{-1}\bar{D}U_{\tau }^{*}\left(\tilde{B}\right)\overset{p}{\to }{𝚺}_{\tau }$ if each column of $\tilde{B}$ converges in probability to $\beta_{0}$,
(III) given all observations, $n^{-1∕2}U_{\tau }^{*}\left(\hat{\beta }\right)\overset{d}{⟶}N\left(0,{𝚺}_{\tau }\right)$ in probability, i.e. it is asymptotically multivariately normally distributed,

if the resampling is done via method Equation (9) or (13).

The next theorem shows that both bootstraps based on the $G_{i}dN_{i}$ approach (Lin et al., 1994) are asymptotically valid. Therein, d denotes a distance that metrizes weak convergence on $R^{p}\times D\left[0,\tau \right]$, e.g. the Prohorov distance (Dudley, 2002, p. 309ff.), and L(T) and L(T∣data) are, respectively, the unconditional and conditional law of a random variable T.

Theorem 1 Under Condition 1 it holds for both resampling strategies Equation (9) or (13) that the asymptotic distributions of $\sqrt{n}\left({\hat{\beta }}^{*}-\hat{\beta },{\hat{\Lambda }}_{0}^{*}-{\hat{\Lambda }}_{0}\right)$ and $\sqrt{n}\left(\hat{\beta }-\beta ,{\hat{\Lambda }}_{0}-\Lambda_{0}\right)$ coincide, that is,

(15) $$d\left[L\left\{\sqrt{n}\left({\hat{\beta }}^{*}-\hat{\beta },{\hat{\Lambda }}_{0}^{*}-{\hat{\Lambda }}_{0}\right)|\text{data}\right\},L\left\{\sqrt{n}\left(\hat{\beta }-\beta ,{\hat{\Lambda }}_{0}-\Lambda_{0}\right)\right\}\right]\overset{p}{\to }0$$

as n→∞, where ${\hat{\Lambda }}_{0}\left(t\right)={\hat{\Lambda }}_{0}\left(t,\hat{\beta }\right)$ and ${\hat{\Lambda }}_{0}^{*}\left(t\right)={\hat{\Lambda }}_{0}^{*}\left(t,{\hat{\beta }}^{*}\right)$.

The asymptotic variance function $t\mapsto \sigma^{2}\left(t\right)$ of $\sqrt{n}\left({\hat{\Lambda }}_{0}-\Lambda_{0}\right)$ (and thus also of $\sqrt{n}\left({\hat{\Lambda }}_{0}^{*}-{\hat{\Lambda }}_{0}\right)$) can be found in Andersen et al. (1993), Corollary VII.2.4, p. 505, where a consistent estimator ${\hat{\sigma }}^{2}\left(t\right)$ is given. In our simulation study in Section 4, we chose the multiplier bootstrap counterpart of ${\hat{\sigma }}^{2}\left(t\right)$ to be the empirical variance of the bootstrap realizations of $\sqrt{n}\left\{{\hat{\Lambda }}_{0}^{*}\left(t\right)-{\hat{\Lambda }}_{0}\left(t\right)\right\}$.

We use the theorem to construct time‐simultaneous confidence bands for $\Lambda_{0}$ on fixed intervals $I=\left[t_{1},t_{2}\right]\subset \left[0,\tau \right]$. In particular, we obtain results similar to those of Lin et al. (1994): denoting by ϕ a continuously differentiable and strictly increasing or decreasing function, asymptotic level 1−α confidence bands for $\Lambda_{0}$ on I are given by

$$\phi^{-1}\left[\phi \left\{{\hat{\Lambda }}_{0}\left(t,\hat{\beta }\right)\right\}\mp c_{\phi }^{*}\left(\alpha \right)∕g_{n}\left(t\right)\right],$$

where $g_{n}:I\to \left(0,\infty \right)$ is a possibly random weight function. Typical choices are

$$g_{n}^{\left(1\right)}\left(t\right)=\sqrt{n}∕\hat{\sigma }\left(t\right)\;\text{and}\;g_{n}^{\left(2\right)}\left(t\right)=\sqrt{n}∕\left\{1+{\hat{\sigma }}^{2}\left(t\right)\right\}$$

in case of the transformation $\phi_{1}\left(x\right)=x$, and

$${\tilde{g}}_{n}^{\left(1\right)}\left(t\right)=\sqrt{n}{\hat{\Lambda }}_{0}\left(t,\hat{\beta }\right)∕\hat{\sigma }\left(t\right)\;\text{and}\;{\tilde{g}}_{n}^{\left(2\right)}\left(t\right)=\sqrt{n}{\hat{\Lambda }}_{0}\left(t,\hat{\beta }\right)∕\left\{1+{\hat{\sigma }}^{2}\left(t\right)\right\}$$

for the transformation $\phi_{2}\left(x\right)=\log\left(x\right)$. The resulting confidence bands correspond to the so‐called *equal precision* (for $g_{n}^{\left(1\right)}$ or ${\tilde{g}}_{n}^{\left(1\right)}$) and *Hall‐Wellner bands* (for $g_{n}^{\left(2\right)}$ or ${\tilde{g}}_{n}^{\left(2\right)}$), respectively. Let $g^{*}\left(t\right)$ and ${\tilde{g}}^{*}\left(t\right)$ be the multiplier bootstrap analogs of $g_{n}^{\left(j\right)}\left(t\right)$ and ${\tilde{g}}_{n}^{\left(j\right)}\left(t\right)$, respectively, j∈{1,2}, that is, we utilized ${\hat{\Lambda }}_{0}^{*}$ instead of ${\hat{\Lambda }}_{0}$ and the corresponding empirical variances instead of $\hat{\sigma }$. We chose $c_{\phi }^{*}\left(\alpha \right)=c_{\phi_{1}}^{*}\left(\alpha \right)$ to be the (1−α) quantile of the conditional distribution of $\underset{t\in I}{\text{sup}}g^{*}\left(t\right)|{\hat{\Lambda }}_{0}^{*}\left(t,\hat{\beta }\right)-{\hat{\Lambda }}_{0}\left(t,\hat{\beta }\right)|$, and the naïve choice for $c_{\phi_{2}}^{*}\left(\alpha \right)$ is the corresponding quantile of $\underset{t\in I}{\text{sup}}{\tilde{g}}^{*}\left(t\right)|\log\left\{{\hat{\Lambda }}_{0}^{*}\left(t,\hat{\beta }\right)\right\}-\log\left\{{\hat{\Lambda }}_{0}\left(t,\hat{\beta }\right)\right\}|$. However, the latter resulted in numerical instabilities, and we preferred the asymptotically equivalent choice $c_{\phi_{2}}^{*}\left(\alpha \right)=c_{\phi_{1}}^{*}\left(\alpha \right)$.

It follows from Theorem 1 that all confidence bands are valid for large sample sizes. To additionally assess their small sample properties, we compare them in Monte‐Carlo simulations in Section 4. There, we also analyze the analogous behavior of the resampling approaches based on $d{\hat{M}}_{i}$. It should be noted that the confidence bands obtained from the ‘classical’ multiplier bootstrap from Section 3.1 with standard normal multipliers are not equal to those proposed in Lin et al. (1994). The key difference is that we additionally use a resampling version of the weight functions (particularly of the variance estimator) to be in line with the usual guidelines for bootstrap‐based inference (Hall & Wilson, 1991). We additionally refer to Allignol et al. (2018), where a similar recommendation was made.

### 3.4 Extensions to more general models and more on inference

A related multiplier bootstrap approach carries over to more general models in multistate set‐ups. In particular, as long as the counting process martingale can be mimicked with the help of bootstrap multipliers, the asymptotics of the resampled estimators result in almost the same way as for the original estimators. Thus, the above methodology can straightforwardly be extended to multistate models with K states and multiplicative intensity processes

(16) $$\lambda_{ih}\left(t,\theta \right)=Y_{ih}\left(t\right)\lambda_{h0}\left(t,\gamma \right)\text{exp}\left\{X\prime_{i}\left(t\right)\beta_{0}\right\}$$

for each transition h=1,…,K(K−1), where $\theta =\left(\gamma ,\beta \prime_{0}\right)\prime$. Different to above, this model allows for an arbitrary number of transitions between different states. Following Bluhmki et al. (2018), the above multiplier bootstrap approach remains applicable: instead of the previously used multipliers $G_{i}$, more general white noise processes ${\left\{G_{ih}\left(u\right)\right\}}_{u}$ with zero mean and unit variance are required. Randomly weighting the counting process increments leads to $G_{ih}\left(u\right)dN_{ih}\left(u\right)$. The evaluation occurs at a finite number of times, so no existence problems arise for the white noise processes, and the implementation is straightforward.

The martingale arguments still hold, so that the multiplier bootstrap mimics the joint stochastic behavior of the parameter and multivariate hazard function estimators. Indeed, according to Bluhmki et al. (2018), independent white noise processes ${\left\{G_{ih}\left(u\right)\right\}}_{u}$ and ${\left\{G_{i\tilde{h}}\left(u\right)\right\}}_{u}$ for transitions $h\neq \tilde{h}$ give rise to orthogonal square‐integrable martingales in $t,\int_{0}^{t}f_{ih}\left(u\right)G_{ih}\left(u\right)dN_{ih}\left(u\right)$ and $\int_{0}^{t}f_{i\tilde{h}}\left(u\right)G_{i\tilde{h}}\left(u\right)dN_{i\tilde{h}}\left(u\right)$, with respect to the filtration

$$F={\left[F_{t}≔\sigma \left\{Y_{ih}\left(u\right),N_{ih}\left(u\right),Y_{i\tilde{h}}\left(u\right),N_{i\tilde{h}}\left(u\right):0\leq u\leq \tau ;\;G_{ih}\left(v\right),G_{i\tilde{h}}\left(v\right):0\leq v\leq t\right\}\right]}_{t\in \left[0,\tau \right]}.$$

Here, $f_{ih}$ and $f_{i\tilde{h}}$ are F‐predictable random functions. The predictable variation process of the first martingale is $\int_{0}^{t}f_{ih}^{2}\left(u\right)dN_{ih}\left(u\right)$, which nicely reflects the properties of the original estimators: the corresponding orthogonal counting process martingales $\int_{0}^{t}f_{ih}\left(u\right)dM_{ih}\left(u\right)$ and $\int_{0}^{t}f_{i\tilde{h}}\left(u\right)dM_{i\tilde{h}}\left(u\right)$ have predictable variation $\int_{0}^{t}f_{ih}^{2}\left(u\right)Y_{ih}\left(u\right)\lambda_{h0}\left(u,\gamma \right)\text{exp}\left\{X\prime_{i}\left(u\right)\beta_{0}\right\}du$, and similarly for $\tilde{h}$. In this sense, the bootstrap‐based predictable variation processes estimate the original predictable variation processes. Extending the proofs in the supporting information based on these findings, large‐sample properties of estimators in multistate problems transfer to their multiplier bootstrap versions in an obvious way, as long as the original estimators admit martingale representations. Similar arguments extend to Cox‐Aalen multiplicative‐additive intensities (Scheike & Zhang, 2002) or other models with martingale structures.

The incorporation of certain filtered (e.g., right‐censored) observations is again allowed and important inferential applications follow: confidence bands for cumulative transition hazards or incidence functions, and tests for null hypotheses formulated in terms of the parameters can be constructed. Here, bootstrap‐based versions of score or Wald‐type test statistics (Martinussen & Scheike, 2006, p. 185f.) might ensure a proper finite sample behavior. However, detailed evaluations of these applications require additional extensive simulations and further elaborations in future research. For simplicity, we continue with our focus on the Cox model Equation (1) and assess the impact of the proposed methods in simulations.

## 4 SIMULATION STUDY

To compare the performances of the various resampling approaches described in Section 3.3, we conducted a simulation study in which we covered situations of small to large sample sizes: n=100,200,400. The generated data follow the Cox survival model with baseline hazard rate $\lambda_{0}\equiv 1$, normally distributed covariates $X_{i}\overset{\text{i.i.d.}}{\sim }N\left(0,16\right)$ with standard deviation σ=4, and regression parameter β=0.3. Censoring times are standard exponentially distributed, truncated at τ=3. The time interval, along which 95% confidence bands for the cumulative baseline hazard function $\Lambda_{0}$ shall be constructed, was $\left[t_{1},t_{2}\right]=\left[0.5,3\right]$. Here we chose the start time of $t_{1}=0.5$ because “the approximations tend to be poor for t close to 0” (Lin et al., 1994, p. 77). Another simulation study that evaluates the multiplier bootstrap for confidence bands for baseline cumulative incidence functions is given in Suporting Information.

As bootstrap multipliers $G_{1},\ldots ,G_{n}$, we considered the common choice $G_{i}\overset{\text{i.i.d.}}{\sim }N\left(0,1\right)$, centered unit Poisson variables $G_{i}\overset{\text{i.i.d.}}{\sim }Poi\left(1\right)-1$ with unit skewness, and centered unit exponential variables $G_{i}\overset{\text{i.i.d.}}{\sim }Exp\left(1\right)-1$ with a skewness of 2. We simulated all confidence bands for $\Lambda_{0}$ that were introduced in Section 3.3, i.e. log‐ and nontransformed Hall‐Wellner and equal precision bands. We considered replacing the martingale increments $dM_{i}$ with $G_{i}dN_{i}$ or $G_{i}d{\hat{M}}_{i}$, denoted in Table 1 as “dN” and “dM”, respectively, and also both kinds of resampling algorithms, the direct resampling method of Section 3.1 and the method of Section 3.2 in which the estimating equations were bootstrapped. We note that, apart from the specific variance estimation scheme, the bands resulting from the cox.aalen function in the R package *timereg* are essentially the same as the multiplier bootstrap from Section 3.1 with no transformation and standard normal multipliers and the results for these are therefore not shown. For each set‐up and type of band, we constructed 10,000 confidence bands, where each was based on 1,000 resampling iterations. The obtained empirical coverage probabilities given in Table 1 are the observed frequencies of constructed bands containing $\Lambda_{0}$ completely.

**Table 1:** Simulated coverage probabilities (in %, rounded) of various 95% confidence bands for the baseline cumulative hazard function and sample sizes n=100,200,400

			Hall‐Wellner				Equal precision			
Distribution of $G_{i}$		Resampling approach	Estimating equation		Direct resampling		Estimating equation		Direct resampling	
	n		id	log	id	log	id	log	id	log
N(0,1)	100	dN	86.8	93.4	87.2	93.4	85.6	92.7	85.5	92.5
		dM	86.9	93.2	87.2	93.6	85.7	92.6	85.2	92.4
	200	dN	90.8	94.2	90.9	94.2	89.8	93.9	89.8	93.8
		dM	90.6	94.0	90.7	94.2	89.4	93.3	89.3	93.2
	400	dN	93.2	95.1	93.3	95.0	92.4	94.1	92.4	94.1
		dM	93.2	94.9	93.2	95.1	92.3	94.1	92.4	94.0
Exp(1)−1	100	dN	88.4	94.4	88.5	94.5	88.8	95.3	89.0	95.3
		dM	88.1	94.3	88.4	94.5	88.7	95.4	87.9	94.6
	200	dN	91.3	94.6	91.3	94.7	91.3	95.4	91.4	95.3
		dM	91.2	94.5	91.2	94.6	91.0	94.9	90.8	94.6
	400	dN	93.6	95.3	93.5	95.2	93.1	94.9	93.1	95.0
		dM	93.9	95.4	94.1	95.5	93.5	95.4	93.4	95.2
Poi(1)−1	100	dN	87.0	93.3	87.1	93.6	86.1	93.3	86.2	93.0
		dM	87.0	93.6	87.4	93.7	86.0	93.2	86.0	92.8
	200	dN	90.6	94.1	90.7	94.2	89.6	93.6	89.7	93.7
		dM	91.0	94.2	91.2	94.3	90.0	94.1	90.0	94.0
	400	dN	93.3	95.0	93.3	95.1	92.4	94.2	92.6	94.2
		dM	93.3	95.1	93.1	95.1	92.4	94.3	92.3	94.2

Distributions of the multipliers $G_{i}$ : standard normal (top panel), centered unit exponential (middle panel), centered unit Poisson (bottom panel)

When the sample size is 400, all methods give a reasonable performance. For smaller samples there are notable differences, and it seems that the log‐transform improves the bands’ performances considerably. Whether the bootstrap is based on $G_{i}dN_{i}$ or $G_{i}d{\hat{M}}_{i}$ does not seem important, and in terms of computations it is considerably easier and faster to use the multipliers based on $G_{i}dN_{i}$. Despite theoretical and practical advantages of Poisson variables over standard normal multipliers in nonparametric competing risks models (Dobler et al., 2017), this choice of bootstrap multipliers does not seem highly important here. However, centered exponential multipliers tend to perform the best. Also, the particular resampling method, be it the direct approach of Section 3.1 or the estimating equation approach of Section 3.2, does not seem to have a clearly positive or negative impact on the outcomes.

**Figure 1:**
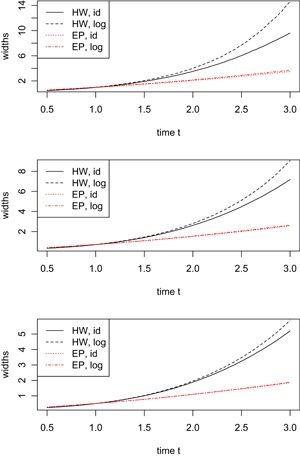
Median widths of untransformed and log‐transformed confidence bands for sample sizes n=100 (top), n=200 (middle), n=400 (bottom panel). Solid and dashed: Hall‐Wellner, pointed and point‐dashed: equal precision. This figure appears in color in the electronic version of this article

Apart from the coverage probabilities, we also computed the bands’ median widths for t∈[0.5,3]. Because of the marginal differences among multiple resampling approaches, Figure 1 only shows the median widths for the $G_{i}dN_{i}$ method with standard normal multipliers, based on resampling the estimating equation. Again, we let n=100,200,400. In all panels the trend is the same: at early time points the equal precision bands are slightly wider than the Hall‐Wellner bands. But for later time points it is the opposite; and the widths of the Hall‐Wellner bands grow rapidly due to quickly increasing variances. Also, the log‐transformed bands are slightly wider than their untransformed counterparts. All in all, we recommend to use log‐transformed equal precision bands for $\Lambda_{0}$ because of their good coverage probabilities (log‐transformation) and their reasonable slimness.

Finally, we remark that our simulation results are only partially comparable to those of Lin et al. (1994): we construct confidence bands for $\Lambda_{0}$, that is, for an individual with $X_{i}=0$; their bands are for survival curves for multiple covariates using also different transformantions.

Overall, however, the bands here and in Lin et al. (1994) seem to work well.

## 5 DATA EXAMPLES

In this section, we illustrate how the resampling approach can be used in two practical settings, based on data that are available in the *timereg* R‐package. The R‐code is given in the supporting information. First, we show how confidence bands can be used and constructed in a standard survival setting. The key point is that a simultaneous confidence band is the type of interval estimate most often of interest unless focus is on a particular survival probability at a specific time such as, for example, 5‐year survival. In addition, we find confidence intervals for derived measures, in particular, for the restricted mean life. The second example illustrates in a competing risks setting how simple it is to combine the multiplier bootstrap based on Cox models for cause‐specific hazards to construct confidence bands for the cumulative incidence.

### 5.1 Survival estimation

We consider the TRACE study (Jensen et al., 1997), where interest is on survival after acute myocardial infarction for 1878 consecutive patients included in the study. The data‐set is available in the *timereg* R‐package. Here for sake of illustration we focus interest on the covariates diabetes (1/0), sex and age. We depict the survival predictions with confidence bands for a male with average age (66.9 years) and with or without diabetes, as well as the standard 95% point‐wise confidence intervals for comparison.

**Figure 2:**
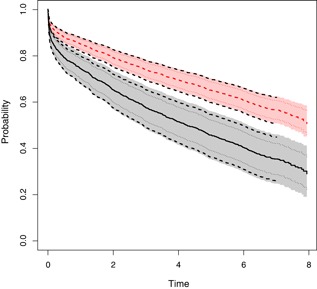
Survival function estimates for males with average age, with (fat broken line) or without diabetes (fat solid line), with 95 % confidence intervals (dotted lines), multiplier bootstrapped log‐transform 95 % confidence bands (broken lines) and 95 % confidence bands of *timereg* (regions). This figure appears in color in the electronic version of this article

We note that the hazard ratio related to diabetes is 1.82 with 95% confidence interval (1.50, 2.18). Thus reflecting that diabetes is a factor that leads to increased mortality. More interestingly, in regard to absolute mortality rates, the higher risk of diabetics is reflected in our estimated survival curves for males with average age and with diabetes (lower set of curves, with confidence bands (region) and point‐wise intervals (dotted lines) or without diabetes (upper set of curves). The regions are standard *timereg*‐bands based on direct resampling and via $G_{i}d{\hat{M}}_{i}$, and for comparison we also show the resampling‐based equal precision bands based on a log‐transform (broken lines). We note a slight difference between the different confidence bands. In addition we note that the bands are quite a bit wider than the point‐wise intervals. As the latter do not provide simultaneous coverage, the bands should be used to provide uncertainty about the entire survival curve as shown in Figure 2.

We finally illustrate how the joint asymptotic distribution of the baseline and parameter estimators can be used with other functionals. To this end, consider the restricted mean life of an individual with lifetime T and covariate vector X, that is,

$$\Psi \left(\Lambda_{0},\beta_{0},X\right)=\int_{0}^{\tau }\text{exp}\left\{-\Lambda_{0}\left(s\right)\text{exp}\left(X\prime \beta_{0}\right)\right\}ds=E\left\{\min\left(T,\tau \right)|X\right\}$$

with estimator $\Psi \left({\hat{\Lambda }}_{0},\hat{\beta },X\right)$. We refer to Karrison (1987) for the concept of adjusting the restricted mean life to covariates. To get a description of the uncertainty of $\Psi \left({\hat{\Lambda }}_{0},\hat{\beta },X\right)$ based on multiplier bootstrap constructions we can simply apply the functional to the obtained bootstrap samples. It follows that $\sqrt{n}\left\{\Psi \left({\hat{\Lambda }}_{0},\hat{\beta },X\right)-\Psi \left(\Lambda_{0},\beta_{0},X\right)\right\}$ has the same asymptotic distribution as $\sqrt{n}\left\{\Psi \left({\hat{\Lambda }}_{0}^{*},{\hat{\beta }}^{*},X\right)-\Psi \left({\hat{\Lambda }}_{0},\hat{\beta },X\right)\right\}$ due to Hadamard differentiability. Thus, we can easily construct symmetric 95% confidence intervals for the restricted mean and their differences based on the bootstrap. The key point being that these are very easy to get at when the bootstrap estimates are at hand. In addition, still due to Hadamard differentiability, differences in restricted mean life $\Psi \left({\hat{\Lambda }}_{0},\hat{\beta },X_{1}\right)-\Psi \left({\hat{\Lambda }}_{0},\hat{\beta },X_{2}\right)$ and uncertainties thereof are also estimable using the obtained bootstrap estimates.

For example, the direct multiplier bootstrap approach based on $G_{i}dN_{i}$ and standard normal multipliers yields that males with diabetes have a restricted mean life within the first 5 years at 3.87 (3.74, 4.00) for males without diabetes and 3.15 (2.91, 3.41) with diabetes. Males with diabetes thus lose 0.71 (0.49, 0.93) years within the first 5 years. Confidence intervals for other functionals are similarly found by means of the functional Δ method.

### 5.2 Cumulative incidence for competing risks

We here consider data on bone marrow transplantation from patients that are treated for myelodysplasia (Sierra et al., 2002). This data is also available in the the *timereg* R‐package. Of 408 patients 161 died from treatment‐related causes, and 87 had relapse before dying, and the remaining were censored. We consider these as two competing risks of death.

We wish to estimate the risk $F_{1}$ of dying from treatment‐related causes by a certain point in time, and do this by modeling the two cause‐specific hazards using Cox regression models based on the covariates platelet count (High 1/Low 0), tcell‐depletion (yes 1/no 0), and standardized age ([age‐35]/15). We then combine the estimates for a particular covariate combination (Low platelets, no tcell‐depletion, and average age (35 years) to estimate the cumulative incidence $F_{1}\left(t|X\right)=\int_{0}^{t}S\left(u|X\right)\lambda_{1}\left(u|X\right)dt$, where S(u∣X) is the probability of surviving both causes, and $\lambda_{1}\left(u|X\right)$ is the intensity for a treatment‐related death.

We denote the baseline cumulative hazards and the parameters of both Cox models by $\theta =\left(\Lambda_{1},\beta_{1},\Lambda_{2},\beta_{2}\right)$. Note that the Cox models for each cause‐specific hazard are resampled independently. Their combination yields inference on the cumulative incidence functional

$$F_{1}\left(\theta \right)\left(t|X\right)=\int_{0}^{t}\text{exp}\left\{-\Lambda_{1}\left(t\right)\text{exp}\left(X\prime \beta_{1}\right)-\Lambda_{2}\left(t\right)\text{exp}\left(X\prime \beta_{2}\right)\right\}\text{exp}\left(X\prime \beta_{1}\right)d\Lambda_{1}\left(dt\right).$$

**Figure 3:**
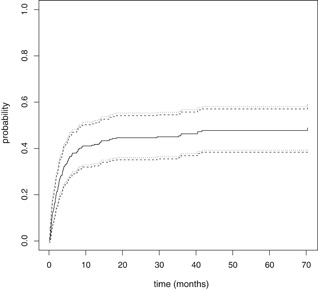
Cumulative incidence curve for a subject with covariate X=0 with 95% equal precision confidence bands with (dotted lines) and without log‐transform (broken lines)

Again, $\sqrt{n}\left\{F_{1}\left(\hat{\theta }\right)-F_{1}\left(\theta \right)\right\}$ has the same asymptotic distribution as $\sqrt{n}\left\{F_{1}\left({\hat{\theta }}^{*}\right)-F_{1}\left(\hat{\theta }\right)\right\}$ due to Hadamard differentiability of the functional. We thus simply need to apply the multiplier bootstrap to the two Cox models and then combine estimates. Subsequently, we can construct a confidence band for $F_{1}\left(\theta \right)$ based on the realizations at hand. Figure 3 shows the cumulative incidence curve for a subject with covariate vector X=0 with 95% equal precision confidence bands with (dotted lines) and without log‐transform (broken lines).

## 6 DISCUSSION AND FURTHER RESEARCH

Despite their importance and considerable interest in making survival predictions based on semiparametric regression models such as the Cox model, confidence bands are not used much in practice. This is probably because the most commonly‐used Cox model routines do not implement simultaneous confidence bands. The aim of this work is to investigate some natural and simple multiplier bootstrap approaches for filling this gap. In particular, we show in the supporting information that the proposed bootstrap solutions do asymptotically have the desired properties. A key point in our proofs is that major characteristics of the procedure can be deduced from martingale arguments. This enormously facilitates the transfer of classical proofs for the estimators to their multiplier bootstrap counterparts. The approach evidently generalizes to more complex models as long as martingale structures for the relevant counting processes are preserved, that is, Cox models in multistate models or Fine‐Gray regression models for subdistribution functions (Fine & Gray, 1999) with administrative censoring. Future work will focus on the adaptation to more complex designs.

In addition, we consider the finite sample performance of various confidence bands. It turns out that the choice of transformation and weight function considerably influence widths and coverage of the bands. Among the bootstrap weights, centered exponential multipliers exhibit the best coverage probability. In particular, their combination with the log‐transformation leads to very accurate results, even for a small sample size (n=100). Furthermore, the equal precision bands exhibit much smaller widths in comparison to the Hall‐Wellner bands.

Another nice feature of our approach is its simple extensibility to find confidence intervals for functionals. We illustrated this with the restricted mean life based on a Cox model.

## Supporting information

Supplementary Information
