# Supplementary material for: Confidence bands for multiplicative hazards models: Flexible resampling approaches
Source: Biometrics. 2019 Apr 17;75(3):906–16. doi: 10.1111/biom.13059 (PMC6849815; doi:10.1111/biom.13059)
Supplement: Supplementary file 1 — Supplementary Information [file BIOM-75-906-s001.pdf]

BIOMETRICS x, 1–13 DOI: ...
August 2018

Supporting Information for
Confidence Bands for Multiplicative Hazards Models: Flexible Resampling Approaches

by Dennis Dobler, Markus Pauly, and Thomas H. Scheike

SUMMARY: In this supporting information, we provide additional information on the algorithmic implementation of the procedures and the corresponding R code, a second simulation study about confidence bands for cumulative incidence functions, as well as the proofs of all theoretical statements.
KEY WORDS: Competing risks; Counting processes; Cox regression; Hazards; Martingale theory; Multi-state models; Survival analysis.

This paper has been submitted for consideration for publication in *Biometrics*

1. Algorithmic Implementation

In the following algorithms we explain the algorithmic application of the bootstrap procedures to construct time-simultaneous confidence bands (TSCBs) of the form

$$\phi^{-1}[\phi\{\widehat{\Lambda}_0(t,\widehat{\boldsymbol{\beta}})\} \mp c^*_\phi(\alpha)/g_n(t)], \quad (1)$$

for the cumulative hazard function Λ_0 on a fixed interval $I = [t_1, t_2] \subset [0, \tau]$ in the Cox Model as described in Section 3.3 of the main paper.

1.1 *TSCBs based on the 'classical' multiplier bootstrap*

We start with the 'classical' multiplier bootstrap based on direct resampling as inspired by Lin et al. (1994) and described in Section 3.1. The Spiekerman and Lin (1998) type versions are obtained by substituting $G_i N_i(t)$ with $G_i\widehat{M_i}(t)$ as described in the manuscript. In the first algorithm we use the untransformed version of (1) based on $\phi = \phi_1 = id$ with the weight function $g_n^{(1)}(t) = \sqrt{n}/\widehat{\sigma}(t)$.

Algorithm 1 The 'classical' multiplier bootstrap – Untransformed TSCBs for Λ_0

Require: Survival data (including covariable vectors) from n subjects

Step 1: Calculate Estimators $\widehat{\boldsymbol{\beta}}, \widehat{\sigma}$ and $\widehat{\Lambda}_0 = \widehat{\Lambda}_0(t,\widehat{\boldsymbol{\beta}})$ from the data.

Step 2: Generate i.i.d. multipliers $G_1, \ldots, G_n \sim N(0,1)$.

▷ (alternatively: $\overset{iid}{\sim}$ $Poi(1)-1$, $Exp(1)-1$ etc.)

Step 3: Calculate $\boldsymbol{U}^*_\tau(\widehat{\boldsymbol{\beta}}), \boldsymbol{I}^*_\tau(\widehat{\boldsymbol{\beta}})$ and $\widehat{\boldsymbol{\beta}}^*$ as defined in Equations (7)–(9)

Step 4: Calculate $\widehat{W}^*(\cdot) := \sqrt{n}\{\widehat{\Lambda}^*_0(\cdot,\widehat{\boldsymbol{\beta}}^*) - \widehat{\Lambda}_0(\cdot,\widehat{\boldsymbol{\beta}})\}$ as defined in Equation (10).

Step 5: Repeat Steps 2–4 B (e.g., $B = 1{,}000$) times and obtain $\widehat{W}^*_1, \ldots, \widehat{W}^*_B$.

Step 6: Calculate $\widehat{\sigma}^*(\cdot)$ as the empirical standard deviation of $\widehat{W}^*_1(\cdot), \ldots, \widehat{W}^*_B(\cdot)$.

Step 7: Calculate $T^*_b = \sup_{t\in I} |\widehat{W}^*_b(t)|/\widehat{\sigma}^*(t)$ for $b = 1, \ldots, B$ and

return $c^*_{\phi_1}(\alpha) = (1-\alpha)$−quantile of $T^*_1, \ldots, T^*_B$.

Step 8: Calculate the TSCB (1) based upon $g_n(t) = g_n^{(1)}(t)$ and $\phi = \phi^{-1} = id$.

Other weight function (untransformed):

If the choice $g_n^{(2)}(t) = \sqrt{n}/\{1+\widehat{\sigma}^2(t)\}$ is preferred, this has to be changed in the preceding steps 7 and 8. To be concrete: Divide by $\{1+\widehat{\sigma}^{*2}(t)\}$ instead of $\widehat{\sigma}^*(t)$ in Step 7 and use $g_n^{(2)}(t)$ instead of $g_n^{(1)}(t)$ in Step 8.

To obtain the log-transformed **equal precision bands** we only have to change the last Step 8 within Algorithm 1:

The weight function $g_n(t) = \tilde{g}_n^{(1)}(t) = \sqrt{n}\widehat{\Lambda}_0(t,\widehat{\boldsymbol{\beta}})/\widehat{\sigma}(t)$ has to be used together with the transformation $\phi(t) = \phi_2(t) = \log(t)$ with $\phi^{-1}(u) = \exp(u)$.

For the log-transformed **Hall-Wellner bands** the same transformation is used together with the weight function $g_n(t) = \tilde{g}_n^{(2)}(t) = \sqrt{n}\widehat{\Lambda}_0(t,\widehat{\boldsymbol{\beta}})/\{1+\widehat{\sigma}^2(t)\}$. Moreover, in Step 7 we have to divide by $\{1+\widehat{\sigma}^{*2}(t)\}$ instead of $\widehat{\sigma}^*(t)$.

As explained in Section 3.3 of the manuscript we also tried to use the $(1-\alpha)$-quantile of $\sup_{t\in I}\tilde{g}^*(t)|\log\{\widehat{\Lambda}_0^*(t,\widehat{\boldsymbol{\beta}})\} - \log\{\widehat{\Lambda}_0(t,\widehat{\boldsymbol{\beta}})\}|$ as critical value $c_\phi^*(\alpha)$ in (1). Due to numerical instabilities, we preferred the current, asymptotically equivalent choice.

1.2 *TSCBs based on bootstrapping the score equations*

We now consider the resampling approach based on bootstrapping the score equations as described in Section 3.2. Again, we start with the untransformed version of (1) based on $\phi = \phi_1 = id$ with the weight function $g_n^{(1)}(t) = \sqrt{n}/\widehat{\sigma}(t)$. The resulting Algorithm 2 is shown below and shares the same first three steps with Algorithm 1.

Algorithm 2 Bootstrapping the score equations – Untransformed TSCBs for Λ_0

Require: Survival data (including covariable vectors) from n subjects

Step 1: Calculate Estimators $\widehat{\boldsymbol{\beta}}, \widehat{\sigma}$ and $\widehat{\Lambda}_0 = \widehat{\Lambda}_0(t, \widehat{\boldsymbol{\beta}})$ from the data.

Step 2: Generate i.i.d. multipliers $G_1, \ldots, G_n \sim N(0,1)$.

▷ (alternatively: $\overset{iid}{\sim} Poi(1) - 1$, $Exp(1) - 1$ etc.)

Step 3: Calculate $\widehat{\Lambda}_0^*(t, \boldsymbol{\beta})$ given in (12).

Step 4: Solve the score equation (13) defined by $\widehat{\Lambda}_0^*(t, \boldsymbol{\beta})$ to find $\widehat{\boldsymbol{\beta}}^*$.

Step 5: Calculate $\widehat{V}^*(\cdot) := \sqrt{n}\{\widehat{\Lambda}_0^*(\cdot, \widehat{\boldsymbol{\beta}}^*) - \widehat{\Lambda}_0(\cdot, \widehat{\boldsymbol{\beta}})\}$ as defined in Equation (14).

Step 6: Repeat Steps 2–5 B (e.g., $B = 1{,}000$) times and obtain $\widehat{V}_1^*, \ldots, \widehat{V}_B^*$.

Step 7: Calculate $\widehat{\sigma}^*(\cdot)$ as the empirical standard deviation of $\widehat{V}_1^*(\cdot), \ldots, \widehat{V}_B^*(\cdot)$.

Step 8: Calculate $S_b^* = \sup_{t \in I} |\widehat{V}_b^*(t)|/\widehat{\sigma}^*(t)$ for $b = 1, \ldots, B$ and

return $c_{\phi_1}^*(\alpha) = (1-\alpha)$−quantile of $S_1^*, \ldots, S_B^*$.

Step 9: Calculate the TSCB (1) based upon $g_n(t) = g_n^{(1)}(t)$ and $\phi = \phi^{-1} = id$.

To obtain the untransformed TSCBs based upon $g_n^{(2)}(t)$ or corresponding log-transformed **equal precision** or **Hall-Wellner bands**, one only has to change the Steps 8 and 9 of Algorithm 2 as described in Subsection 1.1 above for the Steps 7 and 8 of Algorithm 1.

2. R Code

In order to make the developed resampling procedures easily available to all readers, we have decided to upload the R code that has been used in Section 5 in the manuscript. It can be accessed through the following link:

https://github.com/scheike/wildCoxBootstrap

It contains the implementations of all considered resampling procedures and it illustrates how they were used to conduct the real data analyses in Section 5 in the manuscript.

3. Second Simulation Study: Cumulative Incidence in Competing Risks

In addition to the simulation study in Section 4 of the manuscript with a focus on cumulative hazard functions, we conducted a second set of simulations in a competing risks model. In this section we analyze the performance of the multiplier bootstrap in the situation of two competing risks. We focus on confidence bands for the baseline cumulative incidence function of the first risk, $F_1(t \mid X = 0)$. A Cox model with a one-dimensional covariate is used for each cause-specific hazard. The cause-specific baseline hazard rates are $\lambda_1 \equiv \lambda_2 \equiv 0.5$ and we consider two cause-specific hazards of Cox form, $\lambda_1 \exp(X\beta_1)$ and $\lambda_2 \exp(X\beta_2)$, respecitively, where $\beta_1 = 0.3$ and $\beta_2 = -0.3$. The covariates are distributed as $X_i \overset{\text{i.i.d.}}{\sim} \mathcal{N}(0, 16)$ and the right-censoring times follow a mixture distribution of standard exponentially distributed and deterministic times: $C_i \overset{\text{i.i.d.}}{\sim} 0.5Exp(1) + 0.5\varepsilon_3$, where ε_3 is the one-point measure at time $t = 3$. The considered sample-sizes are $n = 100, 200, 400$ as in the main paper.

The aim is again time-simultaneous inference on $F_1(t \mid X = 0) = 0.5(1 - \exp(-t))$ along the time interval $[0.1, 3]$, based on 95% confidence bands. Because of the good performance for simultaneous inference on cumulative baseline hazards, we chose the resampling approach combination of centered standard exponentially distributed multipliers, the estimating equation resampling method, and $G_i \mathrm{d}N_i$. We would like to verify that this choice leads to reliable inference on cumulative incidence functions as well. Table 1 contains the empirical coverage probabilities of the bands which are based on 10,000 independent bands, each of which used 1,000 bootstrap iterations.

[Table 1 about here.]

From the results we can conclude that the multiplier bootstrap again leads to very accurate confidence bands. In particular, also the bands that were not based on the log-transformation showed coverage probabilities close to 95%, even for small sample sizes.

4. Proofs

In the statements of the facts, we always assume that the considered events are measurable which will be the case in all of their applications.

In the following, let $(Z_n)_{n\in\mathbb{N}}$ and $(Z_n^*)_{n\in\mathbb{N}}$ be sequences of random elements in the Skorokhod space $D[0,\tau]$, let Z be a time-continuous random element of $D[0,\tau]$, and let $\mathcal{G}$ be a σ-field with respect to which all Z_n and Z are measurable. Denote by ρ the usual Skorokhod metric.

The first fact which will help us to simplify the proofs in this supporting information is that convergence in probability is equivalent to convergence in conditional probability. This was pointed out to us by a referee who also suggested a proof similar to the one given below, see also Lemma A.5(ii) in combination with Korollar A.4 in Pauly (2009) for a similar argument. This is why we will only refer to unconditional convergence in probability. In particular:

FACT 1: *As $n \to \infty$, $\rho(Z_n, Z) \stackrel{\text{p}}{\to} 0$ if and only if $P(\rho(Z_n, Z) > \varepsilon \mid \mathcal{G}) \stackrel{\text{p}}{\to} 0$ for all $\varepsilon > 0$.*

Proof. Suppose that $\rho(Z_n, Z) \stackrel{\text{p}}{\to} 0$ and define $D_n(\varepsilon) = P(\rho(Z_n, Z) > \varepsilon \mid \mathcal{G})$. By Markov's inequality, for any $\delta > 0$, $P(D_n(\varepsilon) > \delta) \leqslant \delta^{-1}E[D_n(\varepsilon)] = \delta^{-1}P(\rho(Z_n, Z) > \varepsilon) \to 0$.

On the other hand, suppose that $D_n(\varepsilon) \stackrel{\text{p}}{\to} 0$ for every $\varepsilon > 0$. This implies that, for any subsequence $\{n'\}$ of $\{n\}$, there exists a subsequence thereof, say $\{n''\}$, along which $D_n(\varepsilon) \to 0$ P-almost surely. By the dominated convergence theorem, $P(\rho(Z_{n''}, Z) > \varepsilon) = E[D_{n''}(\varepsilon)] \to 0$. Because $\{n'\}$ was arbitrary, $P(\rho(Z_n, Z) > \varepsilon) \to 0$ as $n \to \infty$.

Furthermore, we will make use of a conditional version of Lenglart's martingale inequality (Section II.5.2.1 in Andersen et al., 1993). For completeness, it is stated as another fact.

FACT 2: *Let m be a time-continuous martingale with respect to a filtration $\mathcal{F}$ with a non-trivial initial σ-field $\mathcal{F}_0$. Then, for all $\delta, \eta > 0$,*

$$P(\sup_{0\leqslant s\leqslant\tau} |m(s)| > \eta \mid \mathcal{F}_0) \leqslant \frac{\delta}{\eta^2} + P(\langle m\rangle(\tau) > \delta \mid \mathcal{F}_0).$$

We will rely on martingale arguments for processes of the form

$$m : t \longmapsto \sum_{i=1}^{n} G_i \int_0^t k_{n,\boldsymbol{\beta},i}(s)\mathrm{d}N_i(s).$$

Here the function $k_{n,\boldsymbol{\beta},i}$ is measurable with respect to $\mathcal{F}_0$, which is the non-trivial initial σ-field in the filtration

$$\mathcal{F} = [\mathcal{F}_t = \sigma\{N_i(s), Y_i(s), \boldsymbol{X}_i(s), G_i \cdot \mathrm{d}N_i(v) : 0 \leqslant s \leqslant \tau,\ 0<v \leqslant t,\ i = 1, \ldots, n\}]_{t\in[0,\tau]}.$$

Indeed, it turns out that m is a martingale with respect to $\mathcal{F}$:

Fact 3: *The stochastic process m is a martingale with respect to the filtration $\mathcal{F}$. Its predictable and optional variation process are given by $\langle m\rangle(t) = \sum_{i=1}^n \int_0^t k^2_{n,\boldsymbol{\beta},i}(s)\mathrm{d}N_i(s)$, and $[m](t) = \sum_{i=1}^n G_i^2 \int_0^t k^2_{n,\boldsymbol{\beta},i}(s)\mathrm{d}N_i(s)$, respectively.*

Proof. Consider for $0 \leqslant r \leqslant t$ the conditional expectation

$$E\{m(t) \mid \mathcal{F}_r\} = \sum_{i=1}^{n} G_i \int_0^r k_{n,\boldsymbol{\beta},i}(s)\mathrm{d}N_i(s) + \sum_{i=1}^{n} \int_r^t k_{n,\boldsymbol{\beta},i}(s) E\{Y_i(r)G_i\mathrm{d}N_i(s) \mid \mathcal{F}_r\} = m(r)$$

which is due to $E\{Y_i(r)G_i\mathrm{d}N_i(s) \mid \mathcal{F}_r\} = E\{Y_i(r)G_i\mathrm{d}N_i(s) \mid \mathcal{F}_0\} = E\{G_i \mid \mathcal{F}_0\}Y_i(r)N_i(s) = 0$ for $r \leqslant s$ (Bluhmki et al., 2018). Hence, the martingale property is satisfied. With similar arguments, one finds the stated representations of the predictable and optional variation processes. □

Proof of Lemma 1. To a large extent, it is possible to parallel the martingale arguments as used in the proofs of Theorems VII.2.1 and VII.2.2 in Andersen et al. (1993). We show the proof for the resampling scheme (13) only; once it has been understood how martingale methods can be applied here, it will be apparent how to conduct the proof for the classical multiplier bootstrap scheme (9) which consists of martingales entirely.

Proof of (I). The proof follows the lines of Theorem VII.2.1 in Andersen et al. (1993).

The process $C_t^*(\boldsymbol{\beta}) = \sum_{i=1}^n (G_i + 1)\{\int_0^t \boldsymbol{\beta}'\boldsymbol{X}_i(s)\mathrm{d}N_i(s) - \int_0^t \log S_0(s, \boldsymbol{\beta})\mathrm{d}N_i(s)\}$ satisfies $\nabla C_\tau^*(\boldsymbol{\beta}) = \boldsymbol{U}_\tau^*(\boldsymbol{\beta})$, where ∇ again denotes the gradient with respect to $\boldsymbol{\beta}$. We analyse the

asymptotic behaviour of the process $X^*(t,\boldsymbol{\beta})$

$$= \frac{1}{n}(C_t^*(\boldsymbol{\beta}) - C_t^*(\widehat{\boldsymbol{\beta}})) = \frac{1}{n}\sum_{i=1}^n (G_i+1)\Big\{\int_0^t (\boldsymbol{\beta}-\widehat{\boldsymbol{\beta}})'\boldsymbol{X}_i(s)\mathrm{d}N_i(s) - \int_0^t \log\frac{S_0(s,\boldsymbol{\beta})}{S_0(s,\widehat{\boldsymbol{\beta}})}\mathrm{d}N_i(s)\Big\}$$

whose compensator is $\tilde{X}^*(t,\boldsymbol{\beta}) = \frac{1}{n}\sum_{i=1}^n\{\int_0^t(\boldsymbol{\beta}-\widehat{\boldsymbol{\beta}})'\boldsymbol{X}_i(s)\mathrm{d}N_i(s) - \int_0^t \log\frac{S_0(s,\boldsymbol{\beta})}{S_0(s,\widehat{\boldsymbol{\beta}})}\mathrm{d}N_i(s)\}$; cf. Fact 3. Thus, the martingale $X^*(t,\boldsymbol{\beta})-\tilde{X}^*(t,\boldsymbol{\beta})$ with respect to $\mathcal{F}$ has the predictable variation process

$$\begin{aligned}\langle X^*(\cdot,\boldsymbol{\beta}) - \tilde{X}^*(\cdot,\boldsymbol{\beta})\rangle(t) &= \frac{1}{n^2}\sum_{i=1}^n\int_0^t \Big\{(\boldsymbol{\beta}-\widehat{\boldsymbol{\beta}})'\boldsymbol{X}_i(s) - \log\frac{S_0(s,\boldsymbol{\beta})}{S_0(s,\widehat{\boldsymbol{\beta}})}\Big\}^2\mathrm{d}N_i(s)\\ &= \frac{1}{n^2}\sum_{i=1}^n\int_0^t \Big\{(\boldsymbol{\beta}-\boldsymbol{\beta}_0)'\boldsymbol{X}_i(s) - \log\frac{S_0(s,\boldsymbol{\beta})}{S_0(s,\boldsymbol{\beta}_0)}\Big\}^2\mathrm{d}N_i(s) + O_p(n^{-2}),\end{aligned}$$

where the second equality follows from $\widehat{\boldsymbol{\beta}}-\boldsymbol{\beta}_0 = O_p(n^{-1/2})$ in combination with the mean-value theorem applied to the function $\boldsymbol{\beta}\mapsto \log S_0(s,\boldsymbol{\beta})$, whose gradient, with the different partial derivatives evaluated at different intermediate vectors, is bounded in probability. Standard counting process arguments now reveal that $n\langle X^*(\cdot,\boldsymbol{\beta}) - \tilde{X}^*(\cdot,\boldsymbol{\beta})\rangle(\tau)$ converges for each $\boldsymbol{\beta}$ to a finite number $L(\boldsymbol{\beta})$ and $\tilde{X}^*(\tau,\boldsymbol{\beta}) = \frac{1}{n}\sum_{i=1}^n\Big\{\int_0^\tau(\boldsymbol{\beta}-\boldsymbol{\beta}_0)'\boldsymbol{X}_i(s)\mathrm{d}N_i(s) - \int_0^\tau \log\frac{S_0(s,\boldsymbol{\beta})}{S_0(s,\boldsymbol{\beta}_0)}\mathrm{d}N_i(s)\Big\} + o_p(1)$ to $f(\boldsymbol{\beta}) = \int_0^\tau\Big\{(\boldsymbol{\beta}-\boldsymbol{\beta}_0)'s_1(s,\boldsymbol{\beta}_0) - \log\frac{s_0(s,\boldsymbol{\beta})}{s_0(s,\boldsymbol{\beta}_0)}s_0(s,\boldsymbol{\beta}_0)\Big\}\lambda_0(s)\mathrm{d}s$ in probability as $n\to\infty$. Thus, our next application of Lenglart's inequality will yield that $X^*(\tau,\boldsymbol{\beta}) \stackrel{\mathrm{p}}{\to} f(\boldsymbol{\beta})$ as well: for all $\delta,\eta>0$, we have

$$\begin{aligned}P\Big(\sup_{0\leqslant s\leqslant\tau}\sqrt{n}|X^*(s,\boldsymbol{\beta}) - \tilde{X}^*(s,\boldsymbol{\beta})| > \eta \mid \mathcal{F}_0\Big) &\leqslant \frac{\delta}{\eta^2} + P(n\langle X^*(\cdot,\boldsymbol{\beta}) - \tilde{X}^*(\cdot,\boldsymbol{\beta})\rangle(\tau) > \delta \mid \mathcal{F}_0)\\ &\leqslant \frac{\delta}{\eta^2} + 1\Big\{n\langle X^*(\cdot,\boldsymbol{\beta}) - \tilde{X}^*(\cdot,\boldsymbol{\beta})\rangle(\tau) > \delta\Big\} \quad (2)\end{aligned}$$

due to the $\mathcal{F}_0$-measurability of $n\langle X^*(\cdot,\boldsymbol{\beta}) - \tilde{X}^*(\cdot,\boldsymbol{\beta})\rangle(\tau)$. Now, let $\mathcal{B}_{\boldsymbol{\beta}_0}$ be a neighborhood of $\boldsymbol{\beta}_0$ and choose $\delta > \sup_{\boldsymbol{\beta}\in\mathcal{B}_{\boldsymbol{\beta}_0}} L(\boldsymbol{\beta})$. We thus obtain $P(n\langle X^*(\cdot,\boldsymbol{\beta}) - \tilde{X}^*(\cdot,\boldsymbol{\beta})\rangle(\tau) > \delta) \to 0$. Taking expectations in (2), we get

$$P\Big(\sup_{0\leqslant s\leqslant\tau}\sqrt{n}|X^*(s,\boldsymbol{\beta}) - \tilde{X}^*(s,\boldsymbol{\beta})| > \eta\Big) \leqslant \frac{\delta}{\eta^2} + o(1).$$

Hence, the choice $\eta = \varepsilon\sqrt{n}$ yields $P(\sup_{0\leqslant s\leqslant\tau}|X^*(s,\boldsymbol{\beta}) - \tilde{X}^*(s,\boldsymbol{\beta})| > \varepsilon) \leqslant \frac{\delta}{\varepsilon^2 n} + o(1)$ and thus $|X^*(\tau,\boldsymbol{\beta}) - f(\boldsymbol{\beta})| = |X^*(\tau,\boldsymbol{\beta}) - \tilde{X}^*(\tau,\boldsymbol{\beta})| + |\tilde{X}^*(\tau,\boldsymbol{\beta}) - f(\boldsymbol{\beta})| \stackrel{\mathrm{p}}{\to} 0$.

Now, by Conditions 1(b)–(d), we have $\nabla f(\boldsymbol{\beta}) = \int_0^\tau\{\boldsymbol{e}(s,\boldsymbol{\beta}_0) - \boldsymbol{e}(s,\boldsymbol{\beta})\}s_0(s,\boldsymbol{\beta}_0)\lambda_0(s)\mathrm{d}s$

and $\nabla f(\boldsymbol{\beta}_0) = 0$. Furthermore, $-\nabla^2 f(\boldsymbol{\beta}) = \int_0^\tau \boldsymbol{v}(s,\boldsymbol{\beta}_0)s_0(s,\boldsymbol{\beta}_0)\lambda_0(s)\mathrm{d}s$ which is positive semidefinite and positive definite for $\boldsymbol{\beta} = \boldsymbol{\beta}_0$; cf. Condition 1(e).

Hence, the concave function $f(\boldsymbol{\beta})$ has a unique maximum at $\boldsymbol{\beta} = \boldsymbol{\beta}_0$. The random function $\boldsymbol{\beta} \mapsto X^*(\tau,\boldsymbol{\beta})$ is also concave with a maximum at $\boldsymbol{\beta} = \widehat{\boldsymbol{\beta}}^*$ if it exists. We use Theorem II.1 in Appendix II of Andersen and Gill (1982) to conclude the convergence $X^*(\tau,\boldsymbol{\beta}) \overset{\mathrm{p}}{\to} f(\boldsymbol{\beta})$ which is uniform in neighborhoods of $\boldsymbol{\beta}_0$, as $n \to \infty$. For this reason, the maximizing value $\widehat{\boldsymbol{\beta}}^*$ of $X^*(\tau,\boldsymbol{\beta})$ converges to the maximizing value $\boldsymbol{\beta}_0$ of f in probability. This also holds for $\widehat{\boldsymbol{\beta}}$, hence an applicaiton of Sluztki's lemma yields that $\|\widehat{\boldsymbol{\beta}}^* - \widehat{\boldsymbol{\beta}}\| \overset{\mathrm{p}}{\to} 0$ as $n \to \infty$.

Proof of (II). Recall the definition of $\bar{D}\boldsymbol{U}_\tau^*(\widetilde{\boldsymbol{B}})$ where each column $\boldsymbol{\beta}^{(j)}$ of $\widetilde{\boldsymbol{B}}$ is on the line segment between $\widehat{\boldsymbol{\beta}}^*$ and $\widehat{\boldsymbol{\beta}}$. We again use martingale theory to prove the claimed convergences. Without loss of generality, we assume that $\boldsymbol{\beta}^{(1)} = \cdots = \boldsymbol{\beta}^{(p)}$ because random matrices converge in probability if and only if each row does. Hence, we assume that $\bar{D}\boldsymbol{U}_t^*(\widetilde{\boldsymbol{B}}) = D\boldsymbol{U}_t^*(\boldsymbol{\beta}^{(1)})$ which is the usual Jacobian at $\boldsymbol{\beta}^{(1)}$. The object to be analyzed is thus

$$D\boldsymbol{U}_\tau^*(\boldsymbol{\beta}^{(1)}) = \frac{1}{n}\sum_{i=1}^n (G_i+1)\int_0^\tau \boldsymbol{V}(s,\boldsymbol{\beta}^{(1)})\mathrm{d}N_i(s).$$

We consider the integral until t instead of τ to make use of martingale theory and we apply the decomposition $-n^{-1}D\boldsymbol{U}_t^*(\boldsymbol{\beta}^{(1)}) = A_n(t) + B_n(t) + C_n(t) + D_n(t)$, where

$$\begin{aligned}
A_n(t) =& \frac{1}{n}\sum_{i=1}^n \int_0^t \boldsymbol{V}(s,\boldsymbol{\beta}^{(1)})\mathrm{d}N(s)\\
B_n(t) =& \frac{1}{n}\sum_{i=1}^n G_i\int_0^t \boldsymbol{V}(s,\widehat{\boldsymbol{\beta}})\mathrm{d}N_i(s)\\
C_n(t) =& \frac{1}{n}\sum_{i=1}^n G_i\int_0^t [\{\boldsymbol{V}(s,\boldsymbol{\beta}^{(1)}) - \boldsymbol{v}(s,\boldsymbol{\beta}^{(1)})\} - \{\boldsymbol{V}(s,\widehat{\boldsymbol{\beta}}) - \boldsymbol{v}(s,\widehat{\boldsymbol{\beta}})\}]\mathrm{d}N_i(s)\\
D_n(t) =& \frac{1}{n}\sum_{i=1}^n G_i\int_0^t [\{\boldsymbol{v}(s,\boldsymbol{\beta}^{(1)}) - \boldsymbol{v}(s,\boldsymbol{\beta}_0)\} - \{\boldsymbol{v}(s,\widehat{\boldsymbol{\beta}}) - \boldsymbol{v}(s,\boldsymbol{\beta}_0)\}]\mathrm{d}N_i(s).
\end{aligned}$$

Standard calculus yields

$$|C_n(t)|+|D_n(t)| \leqslant \Big\{2\sup_{s\in[0,\tau],\ \boldsymbol{\beta}\in\mathcal{B}(\boldsymbol{\beta}_0,\delta_n)} |\boldsymbol{V}(s,\boldsymbol{\beta})-\boldsymbol{v}(s,\boldsymbol{\beta})|+|\boldsymbol{v}(s,\boldsymbol{\beta})-\boldsymbol{v}(s,\boldsymbol{\beta}_0)|\Big\}\cdot\frac{1}{n}\sum_{i=1}^n |G_i|N_i(t),$$

where $\mathcal{B}(\boldsymbol{\beta}_0, \delta_n)$ denotes the δ_n-ball around $\boldsymbol{\beta}_0$; $\delta_n = \max(\|\boldsymbol{\beta}^{(1)} - \boldsymbol{\beta}_0\|, \|\widehat{\boldsymbol{\beta}} - \boldsymbol{\beta}_0\|) \in o_p(1)$. Due to the uniform continuity of $\boldsymbol{v}$ in $\boldsymbol{\beta}$ for $s \in [0, \tau]$ (cf. Conditions 1(b,c)), the uniform convergence of $\boldsymbol{V}$ to $\boldsymbol{v}$ in probability as $n \to \infty$ (cf. Conditions 1(a,c)), and $n^{-1}\sum_{i=1}^n |G_i| N_i(t) = O_p(1)$, we conclude that $C_n(t) + D_n(t) = o_p(1)$.

Next, an application of Lenglart's inequality as above shows the asymptotic negligibility of $B_n(t)$ which is a square-integrable martingale in t with respect to $(\mathcal{F}_t)_{t\in[0,\tau]}$.

Finally, $A_n(t)$ is known to converge in probability to $\int_0^t \boldsymbol{v}(s, \beta_0) s_0(s, \beta_0)\lambda_0(s)\mathrm{d}s$; cf. the proof of Theorem VII.2.2 in Andersen et al. (1993). This concludes the proof of (II).

Proof of (III). We will use that $n^{-1/2}\boldsymbol{U}_t^*(\widehat{\boldsymbol{\beta}}) = n^{-1/2}\sum_{i=1}^n G_i \int_0^t \{\boldsymbol{X}_i(s) - \boldsymbol{E}(s, \widehat{\boldsymbol{\beta}})\}\mathrm{d}N_i(s)$ defines a square-integrable martingale in t with respect to $\mathcal{F}$. This can be shown in the same way as for the other martingales above. Its predictable variation process is given by

$$n^{-1}\langle \boldsymbol{U}_{(\cdot)}^*(\widehat{\boldsymbol{\beta}})\rangle(t) = \frac{1}{n}\sum_{i=1}^n \int_0^t \{\boldsymbol{X}_i(s) - \boldsymbol{E}(s, \widehat{\boldsymbol{\beta}})\}^{\otimes 2}\mathrm{d}N_i(s).$$

Similarly as before, this function is asymptotically equivalent to

$$\frac{1}{n}\sum_{i=1}^n \int_0^t \{\boldsymbol{X}_i(s) - \boldsymbol{E}(s, \boldsymbol{\beta}_0)\}^{\otimes 2}\mathrm{d}N_i(s)$$
$$= \frac{1}{n}\sum_{i=1}^n \int_0^t \{\boldsymbol{X}_i(s) - \boldsymbol{E}(s, \boldsymbol{\beta}_0)\}^{\otimes 2}\mathrm{d}M_i(s) + \frac{1}{n}\sum_{i=1}^n \int_0^t \{\boldsymbol{X}_i(s) - \boldsymbol{E}(s, \boldsymbol{\beta}_0)\}^{\otimes 2} S_0(s, \boldsymbol{\beta}_0)\lambda_0(s)\mathrm{d}s.$$

The second term on the right-hand side converges in probability to $\int_0^t \boldsymbol{v}(s, \beta_0) s_0(s, \boldsymbol{\beta}_0)\lambda_0(s)$ while the remaining martingale term vanishes asymptotically: its predictable variation process is given by $\frac{1}{n^2}\sum_{i=1}^n \int_0^t \{\boldsymbol{X}_i(s) - \boldsymbol{E}(s, \boldsymbol{\beta}_0)\}^{\otimes 4} S_0(s, \boldsymbol{\beta}_0)\lambda_0(s)\mathrm{d}s$, where $\mathbf{b}^{\otimes 4}$ is essentially $vec(\mathbf{b}^{\otimes 2})^{\otimes 2}$ for the vectorization $vec(\cdot)$ of matrices. Clearly, this predictable variation goes to zero in probability since the $\boldsymbol{X}_i$ are bounded and the functions $\boldsymbol{E}$ and S_0 converge uniformly in probability to bounded functions. Hence, in order to apply Rebolledo's martingale central limit theorem (Theorem II.5.1 in Andersen et al., 1993) to conclude the asymptotic normality of $n^{-1/2}\boldsymbol{U}_\tau^*(\widehat{\boldsymbol{\beta}})$, only the Lindeberg-type condition remains to be verified. To this end, define for arbitrary $\varepsilon > 0$, the process $t \mapsto n^{-1/2}\boldsymbol{U}_{\varepsilon,t}^*(\widehat{\boldsymbol{\beta}})$ as a cumulation of all jumps of at least

size ε of $n^{-1/2}\boldsymbol{U}^*_{(\cdot)}(\widehat{\boldsymbol{\beta}})$ until t. That is, defining the vector-valued indicator function for each component and the multiplication with it to be component-wise,

$$n^{-1/2}\boldsymbol{U}^*_{\varepsilon,t}(\widehat{\boldsymbol{\beta}}) = \int_0^t 1\{n^{-1/2}\boldsymbol{U}^*_{\Delta u}(\widehat{\boldsymbol{\beta}}) \geqslant \varepsilon\} n^{-1/2}\boldsymbol{U}^*_{\mathrm{d}u}(\widehat{\boldsymbol{\beta}}).$$

Obviously, this process is still a martingale with respect to $\mathcal{F}$, to which we apply Lenglart's inequality: for any $\eta, \delta > 0$, we have, after having taken expectation on both sides as above,

$$P\Big(\sup_{t\in[0,\tau]} |n^{-1/2}\boldsymbol{U}^*_{\varepsilon,t}(\widehat{\boldsymbol{\beta}})| > \eta\Big) \leqslant \frac{\delta}{\eta^2} P(n^{-1}\langle \boldsymbol{U}^*_{\varepsilon,(\cdot)}(\widehat{\boldsymbol{\beta}})\rangle(\tau) > \delta).$$

This predictable variation process is given by $n^{-1}\langle \boldsymbol{U}^*_{\varepsilon,(\cdot)}(\widehat{\boldsymbol{\beta}})\rangle(t)$

$$= \frac{1}{n}\sum_{i=1}^n \int_0^t \{\boldsymbol{X}_i(s) - \boldsymbol{E}(s,\widehat{\boldsymbol{\beta}})\}^{\otimes 2}\, 1\Big\{n^{-1/2}\sum_{i=1}^n G_i\{\boldsymbol{X}_i(s) - \boldsymbol{E}(s,\widehat{\boldsymbol{\beta}})\}\Delta N_i(s) \geqslant \varepsilon\Big\}\mathrm{d}N_i(s).$$

All jump times are assumed to differ. Hence, if any component of the indicator function is one, this implies the existence of i such that $n^{-1/2}|G_i|\|\boldsymbol{X}_i(s) - \boldsymbol{E}(s,\widehat{\boldsymbol{\beta}})\|_\infty \geqslant \varepsilon$. Here, $\|\cdot\|_\infty$ denotes the sup-norm, i.e. the largest absolute value of a vector. We will show that the probability of this event goes to zero across all i and s. To this end, we define the sequence of random variables $Z_n = \sup_{i=1,\dots,n,\ s\in[0,\tau]} \|\boldsymbol{X}_i(s) - \boldsymbol{E}(s,\widehat{\boldsymbol{\beta}})\|_\infty$. From the boundedness assumption on the covariates and the asymptotic behaviour of $\boldsymbol{E}$ and $\boldsymbol{\beta}$, it is clear that Z_n is bounded in probability. That is, there is a constant $K > 0$ such that $P(Z_n > K) \to 0$. Let us consider the probability

$$\begin{aligned} &P(\text{There exist } i,s \text{ such that } n^{-1/2}|G_i|\|\boldsymbol{X}_i(s) - \boldsymbol{E}(s,\widehat{\boldsymbol{\beta}})\|_\infty \geqslant \varepsilon)\\ &\leqslant P\Big(\text{There exists } i \text{ such that } |G_i| \geqslant \frac{\sqrt{n}\varepsilon}{Z_n}\Big)\\ &\leqslant nP\Big(|G_1| \geqslant \frac{\sqrt{n}\varepsilon}{K}\Big) + o(1) = o(1) \end{aligned}$$

where the last equality follows from the existing second moment $E(G_1^2)$ which implies the tail rate $P(|G_1| > t) = o(t^{-2})$ as $t \to \infty$. (This is easily seen from Problem 2.3.3 in van der Vaart and Wellner (1996) because, for all $\varepsilon > 0$, $P(\max_{i=1,\dots,n}|G_i| > \varepsilon\sqrt{n}) \leqslant [\frac{var(G_1)}{\varepsilon^2 n}]^n \to 0$.) All in all, we conclude that the probability that the predictable variation process $n^{-1}\langle \boldsymbol{U}^*_{\varepsilon,(\cdot)}(\widehat{\boldsymbol{\beta}})\rangle$ jumps at all goes to zero. Hence, it converges to zero in probability as $n \to \infty$ and the

Lindeberg-type condition is verified and Rebolledo's martingale central limit theorem applies to $n^{-1/2}\boldsymbol{U}^*_\cdot(\widehat{\boldsymbol{\beta}})$. In particular, it follows that $n^{-1/2}\boldsymbol{U}^*_\tau(\widehat{\boldsymbol{\beta}})$ is asymptotically normal. $\square$

Proof of Theorem 1 We only prove the assertion on the "wild bootstrapping the score equations" approach. The applicability of the Lin et al. (1993, 1994) multiplier scheme follows along the same lines and is in fact easier to prove because less applications of the mean-value theorem are required. All in all, we will make use of similar martingale arguments for the multiplier bootstrapped estimators as in the proof of Lemma 1. To increase readability, some repeating arguments are omitted.

As a first step, paralleling the proof of Theorem VII.2.3 in Andersen et al. (1993), we deduce an asymptotic representation of the first part of $\widehat{\Lambda}^*_0(t,\widehat{\boldsymbol{\beta}}^*) - \widehat{\Lambda}_0(t,\widehat{\boldsymbol{\beta}})$, i.e. of

$$\sum_{i=1}^n (G_i+1)\int_0^t \Big\{\frac{1}{S_0(u,\widehat{\boldsymbol{\beta}}^*)} - \frac{1}{S_0(u,\widehat{\boldsymbol{\beta}})}\Big\}\mathrm{d}N_i(u) = -(\widehat{\boldsymbol{\beta}}^* - \widehat{\boldsymbol{\beta}})\sum_{i=1}^n (G_i+1)\int_0^t \boldsymbol{E}(u,\tilde{\boldsymbol{\beta}}^*)\frac{\mathrm{d}N_i(u)}{S_0(u,\tilde{\boldsymbol{\beta}}^*)}.$$

This equality holds by a Taylor expansion around $\widehat{\boldsymbol{\beta}}$. Here, $\tilde{\boldsymbol{\beta}}^*$ is on the line segment between $\widehat{\boldsymbol{\beta}}^*$ and $\widehat{\boldsymbol{\beta}}$.

Note that we can replace the intermediate $\tilde{\boldsymbol{\beta}}^*$ vectors by $\widehat{\boldsymbol{\beta}}$ because the resulting error

$$\sum_{i=1}^n (G_i + 1)\int_0^t \{\boldsymbol{E}(u,\tilde{\boldsymbol{\beta}}^*)S_0^{-1}(u,\tilde{\boldsymbol{\beta}}^*) - \boldsymbol{E}(u,\widehat{\boldsymbol{\beta}})S_0^{-1}(u,\widehat{\boldsymbol{\beta}})\}\mathrm{d}N_i(u) \tag{3}$$

converges to zero in probability as $n \to \infty$: due to Lemma 1 in combination with Conditions 1(a)–(c), the difference in the curly brackets in (3) is of order $o_p(n^{-1})$ uniformly in s, giving us $\sum_{i=1}^n |G_i + 1| o_p(n^{-1}) = o_p(1)$ as an upper bound of (3). Hence,

$$\sqrt{n}\{\widehat{\Lambda}^*_0(t,\widehat{\boldsymbol{\beta}}^*) - \widehat{\Lambda}_0(t,\widehat{\boldsymbol{\beta}})\} = -\sqrt{n}(\widehat{\boldsymbol{\beta}}^* - \widehat{\boldsymbol{\beta}})\Big\{\sum_{i=1}^n (G_i+1)\int_0^t \boldsymbol{E}(u,\widehat{\boldsymbol{\beta}})\frac{\mathrm{d}N_i(u)}{S_0(u,\widehat{\boldsymbol{\beta}})} + o_p(1)\Big\} + W^*(t),$$

where $W^*(t) = \sqrt{n}\sum_{i=1}^n G_i \int_0^t S_0^{-1}(u,\widehat{\boldsymbol{\beta}})\mathrm{d}N_i(u)$. Of the term in brackets, the sum

$$\sum_{i=1}^n G_i \int_0^t \boldsymbol{E}(u,\widehat{\boldsymbol{\beta}})S_0^{-1}(u,\widehat{\boldsymbol{\beta}})\mathrm{d}N_i(u) = \frac{1}{\sqrt{n}}\Big\{\frac{1}{\sqrt{n}}\sum_{i=1}^n G_i \int_0^t \boldsymbol{E}(u,\widehat{\boldsymbol{\beta}})\frac{n}{S_0(u,\widehat{\boldsymbol{\beta}})}\mathrm{d}N_i(u)\Big\}$$

vanishes because the process inside the curly brackets is a square-integrable martingale with respect to $\mathcal{F}$, and thus asymptotically Gaussian. We conclude that

$$\sqrt{n}\{\widehat{\Lambda}^*_0(t,\widehat{\boldsymbol{\beta}}^*) - \widehat{\Lambda}_0(t,\widehat{\boldsymbol{\beta}})\} = -\sqrt{n}(\widehat{\boldsymbol{\beta}}^* - \widehat{\boldsymbol{\beta}})\Big\{\int_0^t \boldsymbol{e}(u,\boldsymbol{\beta}_0)\lambda_0(u)\mathrm{d}u + o_p(1)\Big\} + W^*(t).$$

It remains to analyze $\sqrt{n}(\widehat{\boldsymbol{\beta}}^* - \widehat{\boldsymbol{\beta}})$ and $W^*(t)$ jointly. Thereof, $\sqrt{n}(\widehat{\boldsymbol{\beta}}^* - \widehat{\boldsymbol{\beta}})$ is essentially a linear transformation of $\boldsymbol{U}^*_\tau(\widehat{\boldsymbol{\beta}})$. Thus, its asymptotic multivariate normality follows from the first two assertions of Lemma 1 in combination with Slutzky's lemma.

For the joint convergence of $\sqrt{n}(\widehat{\boldsymbol{\beta}}^* - \widehat{\boldsymbol{\beta}})$ and $W^*(t)$, we consider the bivariate process $t \mapsto \{n^{-1/2}\boldsymbol{U}^*_t(\widehat{\boldsymbol{\beta}}), W^*(t)\}'$ which, for similar reasons as in the proof of Lemma 1 defines a square-integrable martingale with respect to $\mathcal{F}$. We wish to apply Rebolledo's martingale central limit theorem (with non-trivial initial sigma field $\mathcal{F}_0$) in order to obtain the desired joint conditional central limit theorem which holds in probability. To this end, we analyze the predictable covariation process:

$$\langle n^{-1/2}\boldsymbol{U}^*_{(\cdot)}(\widehat{\boldsymbol{\beta}}), W^*\rangle(t) = \sum_{i=1}^n \int_0^t \{\boldsymbol{X}_i(u) - \boldsymbol{E}(u, \widehat{\boldsymbol{\beta}})\} S_0^{-1}(u, \widehat{\boldsymbol{\beta}}) \mathrm{d}N_i(u). \tag{4}$$

Approximating $\boldsymbol{E}(u, \widehat{\boldsymbol{\beta}})$ on the right-hand side by $\boldsymbol{E}(u, \boldsymbol{\beta}_0)$ (then using (4)) and $S_0^{-1}(u, \widehat{\boldsymbol{\beta}})$ by $S_0^{-1}(u, \boldsymbol{\beta}_0)$, a Taylor expansion around $\boldsymbol{\beta}_0$ and the weak law of large numbers show that (4) is asymptotically equivalent to $\sum_{i=1}^n \int_0^t \{\boldsymbol{X}_i(u) - \boldsymbol{E}(u, \boldsymbol{\beta}_0)\} S_0^{-1}(u, \boldsymbol{\beta}_0) \mathrm{d}N_i(u)$. And this equals

$$\sum_{i=1}^n \int_0^t \{\boldsymbol{X}_i(u) - \boldsymbol{E}(u, \boldsymbol{\beta}_0)\} S_0^{-1}(u, \boldsymbol{\beta}_0) \mathrm{d}M_i(u) \tag{5}$$

$$+ \sum_{i=1}^n \int_0^t \{\boldsymbol{X}_i(u) - \boldsymbol{E}(u, \boldsymbol{\beta}_0)\} S_0^{-1}(u, \boldsymbol{\beta}_0) Y_i(u) \exp\{\boldsymbol{X}_i'(u)\boldsymbol{\beta}_0\} \mathrm{d}\Lambda_0(u). \tag{6}$$

By definition of S_0 and $\boldsymbol{S}_1$, the second term (6) on the right-hand side is zero. The remaining term (5) is a martingale with predictable variation

$$\sum_{i=1}^n \int_0^t \{\boldsymbol{X}_i(u) - \boldsymbol{E}(u, \boldsymbol{\beta}_0)\}^{\otimes 2} S_0^{-2}(u, \boldsymbol{\beta}_0) Y_i(u) \exp\{\boldsymbol{X}_i'(u)\boldsymbol{\beta}_0\} \mathrm{d}\Lambda_0(u)$$
$$= \int_0^t \boldsymbol{V}(t, \boldsymbol{\beta}_0) S_0^{-1}(u, \boldsymbol{\beta}_0) \mathrm{d}\Lambda_0(u) \xrightarrow{\mathrm{p}} 0.$$

Thus, Lenglart's inequality implies that the martingale (5) also goes to zero in probability as $n \to \infty$. Hence, $\sqrt{n}(\widehat{\boldsymbol{\beta}}^* - \widehat{\boldsymbol{\beta}})$ and W^* are asymptotically independent.

Likewise, the other predictable variation processes converge as follows: $\langle n^{-1/2}\boldsymbol{U}^*_{(\cdot)}(\widehat{\boldsymbol{\beta}})\rangle(t) \xrightarrow{\mathrm{p}} \boldsymbol{\Sigma}_t = \int_0^t v(u, \boldsymbol{\beta}_0) s_0(u, \boldsymbol{\beta}_0) \mathrm{d}\Lambda_0(u)$, and $\langle W^*\rangle(t) \xrightarrow{\mathrm{p}} \omega^2(t) := \int_0^t \frac{\mathrm{d}\Lambda_0(u)}{s_0(u, \boldsymbol{\beta}_0)}$ given $\mathcal{F}_0$ in probability

by Conditions 1(a) and (b). Finally, applying Rebolledo's Theorem, it follows that the optional covariation process $\boldsymbol{I}_t^*(\widehat{\boldsymbol{\beta}})$ of $\boldsymbol{U}_t^*(\widehat{\boldsymbol{\beta}})$ converges in probability towards $\boldsymbol{\Sigma}_t$. □

References

PK Andersen and RD Gill. Cox's regression model for counting processes: a large sample study. *The Annals of Statistics*, 10(4):1100–1120, 1982.

PK Andersen, Ø Borgan, RD Gill, and N Keiding. *Statistical Models Based on Counting Processes*. Springer, New York, 1993.

T Bluhmki, C Schmoor, D Dobler, M Pauly, J Finke, M Schumacher, and J Beyersmann. A wild bootstrap approach for the Aalen-Johansen estimator. *Biometrics*, 74(3):977–985, 2018.

C Feng, H Wang, Han Y, Y Xia, and XM Tu. The Mean Value Theorem and Taylor's Expansion in Statistics. *The American Statistician*, 67(4):245–248, 2013.

DY Lin, LJ Wei, and Z Ying. Checking the Cox model with cumulative sums of martingale-based residuals. *Biometrika*, 80(3):557–572, 1993.

DY Lin, TR Fleming, and LJ Wei. Confidence bands for survival curves under the proportional hazards model. *Biometrika*, 81(1):73–81, 1994.

M Pauly. Eine Analyse bedingter Tests mit bedingten Zentralen Grenzwertsätzen für Resampling-Statistiken. PhD thesis University of Duesseldorf, 2009.

AW van der Vaart, and JA Wellner. *Weak Convergence and Empirical Processes.* Springer, New York, 1996.

transformation / n	100	200	400
identity	95.2	94.5	94.5
log	95.0	94.5	94.7

Table 1
Empirical coverage probabilities for the baseline cumulative incidence function $F_1(t \mid X = 0)$, rounded, in %.
